# Short-term memory errors are strongly associated with a drift in neural activity in the posterior parietal cortex

**DOI:** 10.1371/journal.pbio.3003359

**Published:** 2025-09-03

**Authors:** Joon Ho Choi, Sungwon Bae, Jiho Park, Minsu Yoo, Chul Hoon Kim, Lukas Ian Schmitt, Youngbin Tchoe, Dongil Chung, Ji-Woong Choi, Jong-Cheol Rah

**Affiliations:** 1 Sensory and Motor Systems Neuroscience Group, Korea Brain Research Institute, Daegu, South Korea; 2 Department of Pharmacology, College of Medicine, Yonsei University, Seoul, South Korea; 3 Department of Electrical Engineering and Computer Science, Daegu Gyeongbuk Institute of Science and Technology (DGIST), Daegu, South Korea; 4 Center for Brain Science, RIKEN, Wako, Saitama, Japan; 5 Department of Biomedical Engineering, Ulsan National Institute of Science and Technology (UNIST), Ulsan, South Korea; 6 Department of Brain Sciences, DGIST, Daegu, South Korea; Institute of Science and Technology Austria, AUSTRIA

## Abstract

Understanding the neural mechanisms behind short-term memory (STM) errors is crucial for unraveling cognitive processes and addressing deficits associated with neuropsychiatric disorders. This study examines whether STM errors arise from misrepresentation of sensory information or decay in these representations over time. Using 2-photon calcium imaging in the posterior parietal cortex (PPC) of mice performing a delayed match-to-sample task, we identified a subset of PPC neurons exhibiting both directional and temporal selectivity. Contrary to the hypothesis that STM errors primarily stem from mis-encoding during the sample phase, our findings reveal that these errors are more strongly associated with a drift in neural activity during the delay period. This drift leads to a gradual divergence away from the correct representation, ultimately leading to incorrect behavioral responses. These results emphasize the importance of maintaining stable neural representations in the PPC for accurate STM. Furthermore, they highlight the potential for therapeutic interventions aimed at stabilizing PPC activity during delay periods as a strategy for mitigating cognitive impairments in conditions like schizophrenia.

## Abstract 2

What are the neural mechanisms behind short-term memory errors? This study shows that such errors are primarily linked to a drift in neural activity in the posterior parietal cortex, highlighting the need for stable neural representations to support accurate memory performance.

## Introduction

Short-term memory (STM) refers to the capacity to retain immediately relevant information over brief periods, typically on the order of seconds to minutes. While STM is integral to numerous cognitive functions, the maintenance of information is often fragile and prone to errors, frequently leading to suboptimal behavior. Despite the behavioral significance of such errors, the population-level neural dynamics that contribute to them remain poorly understood, particularly in association areas such as the posterior parietal cortex (PPC). Gaining insight into these dynamics is critical for understanding information retention and its implications for psychiatric disorders, including schizophrenia and attention deficit hyperactivity disorder, which are frequently associated with STM deficits [1–3]. This study aims to investigate the population-level neural dynamics underlying STM errors by examining two potential mechanisms that may underlie their generation: (1) Inadequate encoding of information in neural activity during the initial presentation of stimuli, and (2) Disruption of encoded information during the delay period, leading to retention failures.

Along with the prefrontal cortex (PFC), PPC is a critical brain region involved in maintaining information during STM [4–6]. We chose to investigate the activity of the PPC for several reasons. As a major multimodal association cortex, the PPC is ideally positioned to integrate sensory inputs from multiple modalities and relay this accumulated information to prefrontal areas [6,7]. This makes the PPC a plausible origin for erroneous activity during STM error trials, especially if inadequate evidence accumulation or misperception is reflected in both the PFC and PPC. In both primates and rodents, a subset of PPC neurons demonstrates direction-predictive firing rate changes in a graded manner, encoding the clarity of evidence during decision-making tasks such as the random dot motion discrimination task or the Poisson click task [8,9]. In contrast, neurons in prefrontal motor areas show more categorical activity patterns, suggesting that cumulative evidence in the PPC is transformed into discrete decisions within the PFC. Based on these findings, we hypothesized that graded degradation of encoded information, which could lead to STM errors, would be more readily observable in the PPC than in the PFC. By focusing on the PPC, we aimed to capture subtle changes in sensory encoding and retention that might underlie STM errors.

Despite substantial evidence implicating the PPC as a critical substrate for STM, the mechanisms underlying information maintenance in this region remain incompletely understood. In a previous study, mice were trained to navigate a virtual T-maze by choosing a left or right turn based on visual cues presented during the initial phase of each trial, while PPC activity was recorded. Since the visual cue was available only during the first half of the trial, successful navigation required the mice to rely on STM. This study demonstrated that PPC activity involves a complex population encoding, characterized by direction- and phase-selective sequences across multiple neurons [6]. However, interpreting PPC activity during such tasks as a direct reflection of visual STM is challenging due to its navigational attributes [10–12]. For instance, Krumin and colleagues [13] demonstrated that sequential PPC activity during a similar behavioral task could be accurately predicted by the combination of the animal’s heading direction and spatial location within the virtual corridor. Further evidence of task [13]-relevant PPC activity comes from mice performing a visually guided delayed go/no-go task [6,14]. In this paradigm, the PPC exhibited distinct activity patterns, with a substantial number of neurons selectively encoding ‘go’ signals, while ‘no-go’ signals were represented more weakly [6,14]. This observation raises important questions about whether these activities genuinely reflect the retained visual signal or are more related to the suppression and initiation of impulsive movement [15].

To more directly investigate how sensory information is retained during the delay period and how disruptions in this process contribute to errors, we recorded PPC activity while mice performed a head-fixed, delayed match-to-sample task. Mice were presented with a brief lateralized visual stimulus and, after a delay, were required to report the stimulus direction by licking either the left or right port. Crucially, the task did not involve navigation or movement across space, allowing us to isolate memory-related activity. A substantial fraction of imaged PPC neurons exhibited direction-selective and temporally ordered activity during the delay period, despite the absence of navigation. Notably, these patterns closely resembled those observed in previous STM studies [6], supporting the idea of a shared mechanism for encoding short-term representations across different tasks. A comparison of PPC activity between correct and incorrect trials revealed key differences in the neural dynamics underlying STM errors. Specifically, errors were associated with weak initial encoding of the sensory signal, followed by spontaneous drift of neural activity toward an incorrect representation during the delay period. These findings shed light on the population-level dynamics underlying STM errors, providing critical insight into the processes of sensory encoding and maintenance. Additionally, this work opens avenues for future research into how STM mechanisms may be disrupted in neuropsychiatric disorders.

## Results

### Sequence of choice-dependent neuronal activity in PPC

**Fig 1:**
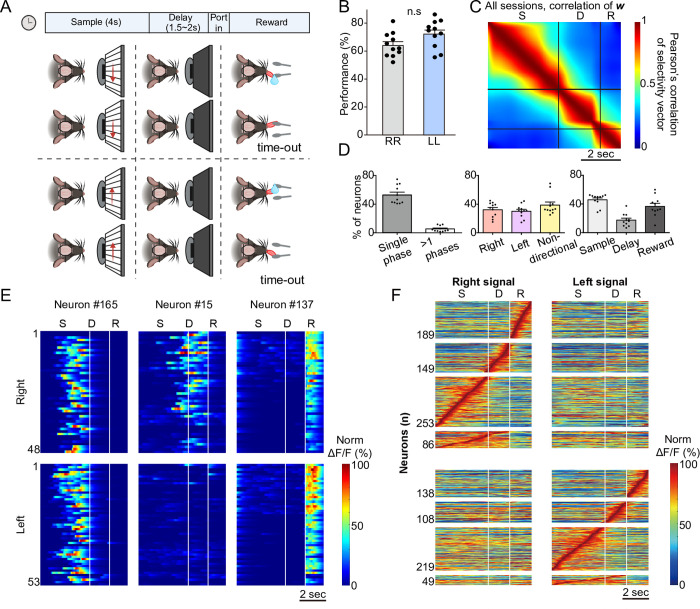
Ca^2+^ responses of PPC neurons during a short-term memory task. **A.** The schematic illustrates the behavioral task designed to assess short-term memory in head-restrained mice. During the sample phase, black-and-white phase-reversing gratings moved either leftward or rightward (or downward or rightward) for 4 s on a screen in front of the mouse. After a 1.5- or 2-s delay, two laterally positioned lick ports were placed within reach of the mouse’s tongue. Correct licking at the corresponding lick port was rewarded with water, whereas incorrect licking resulted in a time-out as a punishment. **B.** Performance comparison between rightward and leftward trials in well-trained mice. No significant difference was observed (two-tailed paired *t* test; *n* = 11, *p* < 0.05). Error bars represent the standard error of the mean (SEM) across sessions. n.s: not significant. **C.** The color-coded autocorrelation matrix of mean response vectors, ***w***, was calculated from the coding direction (CD) of correct trials. ***w*** was calculated over moving 0.5-s time windows. Data represent 11 sessions, 6 mice, and 4,741 total neurons. S: sample phase, D: delay phase, and R: reward phase. **D.** Proportion of neurons displaying task-dependent activity based on a GLM. Left Panel: fraction of neurons with single-phase selectivity, and neurons active in two or more phases as a percentage of all recorded neurons. Middle Panel: percentages of single-phase selective neurons with and without directional selectivity. Right Panel: phase selectivity distribution among single-phase selective neurons. **E.** Representative neuronal activity recorded during the behavioral tasks. Each row shows the color-coded normalized **Δ***F*/*F*. **F.** Color-coded trial-averaged Δ*F*/*F* traces of direction- and phase-selective neurons. Left Panel: neurons selective for rightward stimuli; Right Panel: neurons selective for leftward stimuli. Δ*F*/*F* values were normalized to the maximum Δ*F*/*F* and aligned based on each neuron’s time-to-peak response. Within each directional group, neurons are subdivided by phase selectivity based on GLM classification: those selective for the sample (S), delay (D), or both phases. The bottom panels display neurons that were jointly selective for both sample and delay phases (86 for rightward, 49 for leftward). Underlying data and analysis code for this figure are available at DOI: https://doi.org/10.5281/zenodo.15872574.

Our objective was to determine the extent to which PPC activity is critical for maintaining sensory information during an STM-dependent perceptual decision-making task, excluding confounding factors such as spatial location and head direction. To this end, we trained seven transgenic mice expressing GCaMP6f (C57BL/6J-Tg(Thy1-GCaMP6f)GP5.5Dkim/J) to perform STM tasks. In this task, head-restrained mice were presented with moving gratings displayed on a screen in front of them, with the direction of motion (leftward or rightward) determined pseudo-randomly. After the visual stimulus, there was a 1.5- or 2 s delay during which time no visual input was provided. Following this delay, a bifurcated lick port was introduced within the mice’s reach. Correct responses, consisting of licking the port corresponding to the motion direction of the grating, were rewarded with approximately 5 μL of water. Incorrect responses, defined as licking the opposite port, triggered an extended inter-trial interval of 2 s as a “time-out” penalty (see Fig 1A). After extensive training, mice reached a stable performance level of approximately 70%, which provided a behavioral plateau while allowing us to sample sufficient error trials for analysis. Importantly, the mice displayed no directional bias following training, indicating reliable task learning and unbiased decision-making (Fig 1B).

During task performance, we recorded the activity of PPC neurons by analyzing fluorescence changes in the GCaMP6f-expressing layer (L) 2/3 neurons using two-photon laser scanning microscopy. The identification of the PPC region was guided by stereotaxic coordinates validated through mesoscopic mapping study [16]. Active neurons were identified through post hoc analysis using the CaImAn software [17]. We observed a variety of neuronal responses within the PPC; however, a substantial proportion of neurons consistently exhibited selectivity for direction, task phase, or both with response patterns that were stable across trials (Fig 1E). In addition to the striking consistency of these responses across trials, we computed the time-wise correlation of mean response vectors across all recorded neurons using a sliding window of 15 frames (~0.5 s) with a step size of 1 frame across. This analysis revealed that neural activity was consistent within behavioral phases, further supporting the robustness of PPC encoding during task performance (Fig 1C; see Materials and methods).

To objectively assess task-related selectivity across PPC neurons, we employed a modified generalized linear model (GLM), as described in the Materials and methods section [18]. This approach was motivated by three considerations. First, we aimed to identify whether individual neurons exhibited significant selectivity for specific task phases or stimulus directions, which could be obscured in population-level analyses. Second, we sought to enable direct comparison with prior work [6,19] that used neuron-level classification to reveal structured activity sequences in PPC during navigation tasks. Third, many PPC neurons in our data displayed temporally confined activity—selective for either the sample or delay phase—making direction selectivity less pronounced in global population decoding. The GLM allowed us to resolve these temporally structured patterns in an unbiased and statistically principled manner while accounting for trial-by-trial variability. We used a GLM to classify neurons based on their activity selectivity for distinct task phases (sample, delay, or reward) and stimulus directions, providing a structured categorization of task-related neural responses (See Materials and methods and S15 Fig).

To validate the robustness of this classification, we tested whether GLM-defined phase selectivity was consistently expressed at the single-trial level. For each neuron identified as phase-selective, we computed the difference in Δ*F*/*F* between the preferred phase and the nonpreferred phases on a trial-by-trial basis, and converted these differences into Z-scores relative to a null distribution generated by phase-label permutation (S5 Fig). Phase-selective neurons exhibited a significant bias toward high Z-scores, indicating consistent trial-level selectivity. In contrast, neurons not classified as phase-selective showed a Z-score distribution skewed toward negative values, supporting the specificity of the GLM classification (S5C Fig). Notably, some neurons with weaker Z-scores in this analysis were later found to encode additional variables (e.g., stimulus direction). In addition to the Z-score analysis, we also quantified the consistency of phase-specific firing across trials by computing a one-tailed *p*-value threshold (*p* < 0.05) from the null distribution. We then calculated the proportion of trials in which the observed phase-specific Δ*F*/*F* value exceeded this threshold. This percentage was used as a measure of the reliability of phase-specific responses on a per-trial basis and visualized across the neuronal population (S5B Fig).

Furthermore, the time-correlation of activity vectors from GLM-classified neurons was nearly identical to that of all recorded neurons (S2 Fig), suggesting that the phase-dependent feature of the activity is well-preserved. Our analysis revealed that nearly half of the active neurons in the PPC exhibited selectivity for either phase or stimulus direction (*p* < 0.01), totaling 2,664 out of 4,741 neurons (S3 Fig). Of these, ~60% (1,609/2,664 neurons; 873 for the right signal and 736 for the left signal) exhibited directional selectivity, while the remaining neurons responded similarly to both directions (S4A–S4C Fig). Among the 1,609 directionally selective neurons, ~ 41% displayed phase selectivity, with 661 neurons showing phase selectivity during the sample phase and 315 during the delay phase. Consistent with previous studies, we found that PPC neurons rarely exhibited sustained activity across multiple phases of the task [6], with less than 8.8% (234/2664) of neurons demonstrating phase selectivity during both the sample and delay phases (Figs 1D and S5A).

**Fig 2:**
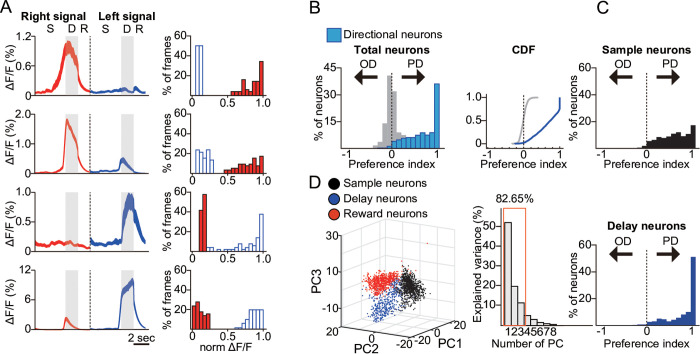
Identification and characterization of the direction- and phase-selective neurons. **A.** Left Panel: example traces and a Right Panel: histogram of trial-averaged Δ*F*/*F* from highly directional phase-selective neurons in the posterior parietal cortex (PPC). The thickness of the traces indicates the standard error of the mean (SEM). Blue and red in the histogram represent leftward and rightward trials, respectively. **B.** Left Panel: Histogram of preference indices (PI) for General Linear Model (GLM)-defined directional neurons (light blue) compared with those calculated from shuffled trials (gray). Right Panel: Cumulative distribution function (CDF) of PI values for the same data. The distributions of the actual data and the randomly shuffled data were significantly different (two-sample Kolmogorov–Smirnov test, *D* = 0.876, *p* < 0.001). **C.** PI histograms of directional neurons during Upper Panel: the sample phase and Lower Panel: delay phase (lower). **D.** Principal components analysis (PCA) capturing the temporal activity patterns of individual neurons. GLM-defined selectivity for the sample (black), delay (blue), or reward (red) phase is indicated by colors. The variance explained by the principal components is also shown. Underlying data and analysis code for this figure are available at DOI: https://doi.org/10.5281/zenodo.15872574.

When the neurons were sorted based on the onset of peak responses, a clear directional-selective sequence of neuronal activity emerged, closely resembling the findings of Harvey and colleagues [6] (Fig 1F). This result underscores the replicability of the directional-selective neural sequence in the PPC, even within a behavioral paradigm that excluded spatial navigation. To move beyond this descriptive observation, we next aimed to quantify the strength and reliability of these direction-selective responses across the neuronal population. We used a receiver operating characteristic (ROC) analysis to verify direction selectivity at the single-neuron level, and introduced the preference index (PI) as a quantitative measure of direction bias based on trial-averaged Δ*F*/*F* responses during the phase of interest (e.g., sample or delay) [20], calculated as hyperbolic area of the ROC curve ((*auROC* − 0.5) × 2). This was complemented by the reliability index (RI), which applies the same analysis to single-trial Δ*F*/*F* values to assess how consistently a neuron distinguishes between directions across trials (see Materials and methods). These metrics confirmed that a substantial fraction of PPC neurons, particularly those active during the delay period, exhibited highly selective and reliable responses to visual direction. Notably, more than 30% of direction-selective neurons showed PI values greater than 0.9, indicating near-perfect separation of responses (Fig 2A and 2B. Furthermore, the higher RI in delay-phase neurons suggested that encoded information becomes more refined after stimulus offset (S4 Fig) [20]. Lastly, principal component analysis (PCA), which captures the temporal activity patterns of individual neurons, demonstrated that neurons with different phase selectivity clustered into distinct low-dimensional representations (Fig 2D). This finding reinforces the validity of our GLM-based classification and provides a structured view of population-level dynamics. Together, these analyses establish that the observed sequences are not only visually salient but also statistically robust, laying the groundwork for downstream comparisons between correct and error trials.

To determine whether the observed activity patterns were organized anatomically, we further assessed the relative co-localization of PPC neurons with similar response properties. Specifically, we compared the distances within groups of neurons with shared functional characteristics to those across groups (S6 Fig). Consistent with previous findings from STM activity in navigation tasks [6], we found no significant difference in the distances to functionally similar neurons compared to those with distinct firing patterns. This refutes the hypothesis that activity patterns are specifically localized. In summary, our results support the idea that activity sequences in the PPC can encode STM of sensory input, even in a delayed match-to-sample task that does not require the encoding of heading direction or spatial location.

### Functional characteristics of directional neurons in error trials

Having established that PPC neurons exhibit direction- and phase-selectivity independent of spatial navigation, we next sought to investigate the mechanisms underlying the disruption of PPC activity during error trials. Specifically, we aimed to determine whether errors in STM could be attributed to inadequately encoded sensory inputs during the sample phase, poor retention of the encoded information during the delay phase, or a combination of both.

**Fig 3:**
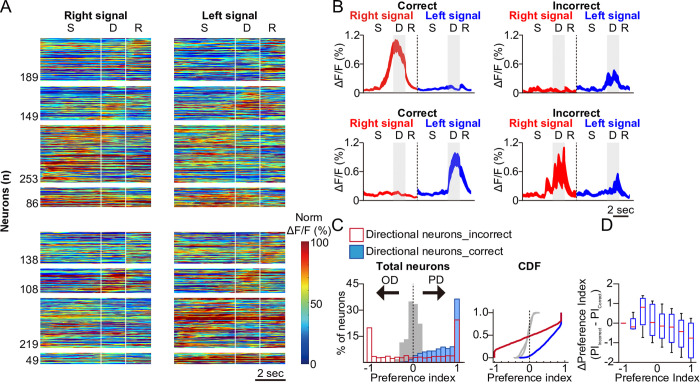
Responses of the direction- and phase-selective neurons in error trials. **A.** Heatmaps of trial-averaged Δ*F*/*F* activity of direction- and phase-selective neurons during error trials, displayed in the same format as Fig 1F. Left panels: neurons selective for rightward stimuli; Right panels: neurons selective for leftward stimuli, further subdivided by phase selectivity (sample [S], delay [D], or both) as classified by the GLM. Δ*F*/*F* traces were normalized to each neuron’s peak response during correct trials and are displayed in the same order as in Fig 1F. Bottom panels show neurons jointly selective for both sample and delay phases (86 for rightward, 49 for leftward). **B.** The mean Δ*F*/*F* traces in error trials of the same example neurons shown in the first and third row in Fig 2A. **C.** Left Panel: Histogram of preference indices (PI) of GLM-defined directional neurons in correct (light blue), error (red open), and shuffled (gray) trials. Right Panel: Cumulative distribution function (CDF) of the left histogram. The distributions of PI values for correct and error trials among the sorted neurons were significantly different (two-sample Kolmogorov–Smirnov test, *D* = 0.446, *p* < 0.001). **D.** A box plot illustrating the difference of PIs (ΔPI) between error and correct trials for each neuron, as a function of PIs in correct trials. Underlying data and analysis code for this figure are available at DOI: https://doi.org/10.5281/zenodo.15872574.

**Fig 4:**
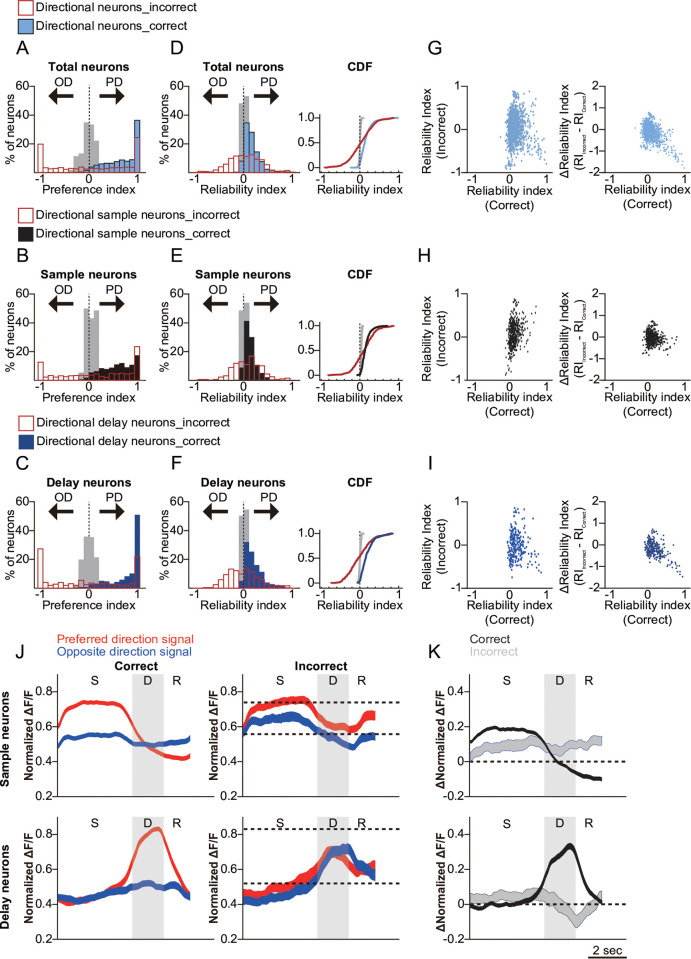
Comparison of responses between delay phase-selective neurons and sample phase-selective neurons during error trials. **A.** Histogram of preference indices (PI) for GLM-defined directional single-phase selective neurons during correct trials (light blue), error trials (red open), and shuffled trials (gray). **B.** Histogram of PI values for directional sample-phase selective neurons during correct trials (black), error trials (red open), and shuffled trials (gray). **C.** Histogram of PI values for directional delay-phase selective neurons during correct trials (blue), error trials (red open), and shuffled trials (gray). **D.** Left Panel: histogram of reliability indices (RI) for GLM-defined directional single-phase selective neurons during correct trials (light blue), error trials (red open), and shuffled trials (gray). Right Panel: cumulative distribution function (CDF) of the RI values from the left histogram. The distributions of RI values for correct and error trials among the sorted neurons were significantly different (two-sample Kolmogorov–Smirnov test, *D* = 0.399, *p* < 0.001). **E.** Left Panel: RI histogram for directional sample-phase selective neurons during correct trials (black), error trials (red open), and shuffled trials (gray). Right Panel: of the RI values from the left histogram. The distributions of RI values for correct and error trials among the sample-phase selective neurons were significantly different (two-sample Kolmogorov–Smirnov test, *D* = 0.352, *p* < 0.001). **F.** Left Panel: RI histogram for directional delay-phase selective neurons during correct trials (blue), error trials (red open), and shuffled trials (gray). Right Panel: CDF of the RI values from the left histogram. The distributions of RI values for correct and error trials among the delay-phase selective neurons were significantly different (two-sample Kolmogorov–Smirnov test, *D* = 0.498, *p* < 0.001). **G.** Left Panel: scatter plot comparing the reliability index RI in correct and incorrect trials for GLM-defined directional neurons in PPC. RI values close to 1 during “correct” trials indicate significant responses in the preferred direction, while values close to −1 during “incorrect” trials suggest responses closer to the opposite direction. Right Panel: scatter plot comparing the RI during correct trials with the difference in RI between incorrect and correct trials. ΔRI values closer to −1 indicate that responses during incorrect trials were closer to the opposite direction compared to correct trials. **H.** Left Panel: scatter plot comparing the RI of GLM-defined directional sample-phase selective neurons in the PPC during correct and incorrect trials. Right Panel: scatter plot comparing the RI (Correct) of GLM-defined direction-selective sample neurons with the difference between the reliability indices for incorrect and correct trials. **I.** Left Panel: scatter plot comparing the RI of GLM-defined directional delay-selective neurons in the PPC during correct and incorrect trials. Right Panel: scatter plot comparing the RI (Correct) of GLM-defined direction-selective delay neurons with the difference between the reliability indices for incorrect and correct trials. **J.** Mean normalized Δ*F*/*F* of direction-selective neurons, separated by phase selectivity. Upper Panel: sample-phase selective neurons during correct (left) and incorrect (right) trials, showing responses to preferred (red) and opposite (blue) directional stimuli. Lower Panel: same as the upper but for delay-phase selective neurons. **K.** Difference between preferred and opposite direction responses (Δ normalized Δ*F*/*F*) of direction-selective neurons. Upper Panel: Δ mean normalized Δ*F*/*F* of direction-selective sample-phase neurons during correct trials (black) and incorrect trials (gray), calculated by subtracting the value of the opposite directional signal response from the preferred directional signal response. Lower Panel: same as above, but for delay-phase selective neurons. Underlying data and analysis code for this figure are available at DOI: https://doi.org/10.5281/zenodo.15872574.

The activity of selective PPC neurons identified during correct trials exhibited markedly lower phase- or direction-selectivity during error trials (Fig 3A). On an individual neuron level, we frequently observed that neurons with high PIs exhibited opposite directional selectivity during error trials (Fig 3B). It is important to note that not all directionally selective neurons responded in the opposite direction, suggesting a mixed population response with some degree of mis-encoding in STM. There was a significant reduction in PI during incorrect trials, particularly among neurons that exhibited high PI during correct trials (Fig 3C). In fact, the magnitude of PI reduction was greater for neurons that had high PI in correct trials (Figs 3D and S7). The median ΔPI for neurons with an initial PI between 0.9 and 1 was approximately −1, indicating that nearly 50% of the highly reliable neurons either lost response selectivity or responded to the opposite direction during error trials. To assess how these activity changes were related to the consistency of responses across individual trials, we examined the trial accuracy-dependent difference in RI values for phase- and direction-selective neurons as well as nondirection-selective neurons (Figs 4A-I and S7). This analysis revealed that a greater proportion of sample-phase neurons maintained directional information regardless of trial accuracy, while delay-phase neurons exhibited increased activation in the opposite direction during error trials. Notably, highly reliable directional delay neurons exhibited the greatest reductions in RI during error trials (Fig 4G–4I). Together, these results suggest that highly reliable directional delay neurons are more likely to show a response pattern opposite to that observed during correct trials.

A reversal in firing responses for neurons with high selectivity would predict a diminished overall difference between preferred and nonpreferred directions. Indeed, we observed a significant reduction in the difference between Δ*F*/*F* of preferred and opposite directional cues during error trials (Fig 4J). This reduced difference was the most pronounced among delay-selective neurons. Specifically, we noted a decrease in Δ*F*/*F* in the preferred direction and an increase in Δ*F*/*F* in the opposite direction, effectively rendering the two responses during the delay period indistinguishable (Fig 4K). These findings suggest that the response selectivity of delay neurons was more profoundly affected than that of sample neurons during error trials.

### Activity trajectory distances of the direction-selective PPC neurons during the task

The observation that a subset of direction-selective neurons exhibited activity patterns corresponding to their response to the opposite directional signal during error trials suggests the possibility of either mis-encoding or disrupted retention of STM information. To directly compare the contributions of these two potential mechanisms, we next examined the temporal changes in the population-wide activity of directional neurons. Specifically, we aimed to determine whether the STM-associated population dynamics initially resembled the opposite direction during the sample phase, gradually shifted towards the opposite direction as the trial progressed, or reflected both patterns.

**Fig 5:**
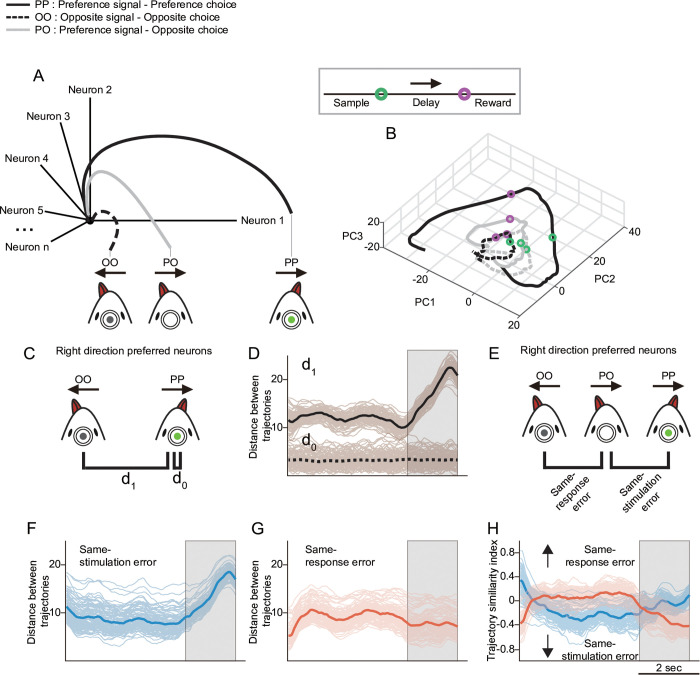
Differences in population activity of direction-selective neurons in correct and error trials. **A.** Schematic representation of the dimensionality reduction approach used to assess differences in population activity. **B.** Population activity trajectories of direction-selective neurons in response to preferred (solid line) and opposite (dashed line) directional stimulation during correct (black) and incorrect (gray) trials. The trajectories were visualized in a 3-dimensional space for clarity, although analyses were conducted in a 5-dimensional subspace. Eigenvectors were generated using 50% of correct trials (randomly chosen) and the remaining correct trials and error trials were then projected onto these eigenvectors. Faint lines represent values from 100 random iterations. **C.** Schematic comparing trajectories of correct preferred-direction responses (PP) and correct opposite-direction responses (OO) for each neuron. **D.** Temporal evolution of the Euclidean distance between PP and OO trajectories (d1). For comparison, d0 represents the trial-by-trial activity variation, measuring the distance between a randomly selected preferred direction response and other preferred direction responses within correct trials. **E.** Schematic comparing trajectories of error trials in which, following a preferred‐direction stimulation, an opposite‐direction response occurs (PO), relative to trajectories of correct preferred‐direction (PP) and correct opposite‐direction (OO) responses for each neuron. **F.** Same-stimulation error: distance between trajectories of correct preferred-direction responses (PP) and incorrect preferred-direction responses (PO). **G.** Same-response error: Euclidean distance between trajectories of correct opposite direction responses (OO) and incorrect preferred-direction responses (PO). **H.** Temporal dynamics of the trajectory similarity index for same-stimulation error (blue) and same-response error (orange). The trajectory similarity index captures the degree of overlap between trajectories in the reduced-dimensional subspace (see S9D Fig for additional details). Responses of neurons with preference opposite to the stimulus direction are shown in S10 Fig. Underlying data and analysis code for this figure are available at DOI: https://doi.org/10.5281/zenodo.15872574.

To this end, we first captured the dynamics of direction-selective neurons in a reduced dimension by representing their activity trajectories through PCA. PCA was applied to trial-averaged calcium activity during correct trials, using neurons aligned to their preferred stimulus direction (see Materials and methods). The top five principal components, which together accounted for over 70% of the total variance, were used to define the low-dimensional neural trajectory space for subsequent analyses. To simplify comparisons, we use two-letter codes, where the first letter denotes the stimulus direction, and the second letter denotes the behavioral response direction (P = preferred, O = opposite). For example, “PO” refers to trials with a preferred-direction stimulus but an opposite-direction (i.e., incorrect) response. The activity trajectories corresponding to correct-preferred direction responses (specifically, the left trial responses from left-preferring neurons and the right trial responses from right-preferring neurons) were designated as preferred-directional correct responses, or PP. In contrast, the trajectories corresponding to correct opposite-directional responses (i.e., right trial responses from left-preferring neurons and left trial responses from right-preferring neurons) were designated as opposite directional responses, or OO (Figs 5 and S9, S10). As a baseline for population variability, we computed the distance between randomly selected PP trials and the remaining PP trials, denoted as d0. The trajectory distance between PP and OO (d1) remained consistently high throughout the sample period and further increased during the delay period (Fig 5C, d1), reflecting a growing divergence in population activity.

To assess how population activity changes in error trials, we next measured the distance from error trials to both PP and OO trajectories. We first compared population activity in error trials to that of correct trials with the same stimulus direction (i.e., PP versus PO). These ‘same-stimulation’ distances (Fig 5F, same-stimulation error) dropped slightly during the sample period, but remained significantly greater than d0, the within-PP variability (two-tailed unpaired *t* test; *n* = 100, *p* < 0.001; S9B Fig). The divergence then increased markedly during the delay phase. We next compared error trials to correct trials that shared the same behavioral response (i.e., ‘same-response’ comparisons, i.e., OO versus PO). These distances rose rapidly during the early sample phase and saturated within the first 0.5 s. However, they remained significantly lower than the PP–OO distance (d1), suggesting that stimulus representations were less distinct in error trials (*p* < 0.001, S9C Fig). During the delay phase, these distances gradually declined (gray area, Fig 5G), suggesting that population activity gradually shifted toward the incorrect response representation.

However, since the error activity trajectories may not necessarily lie within the same activity plane as the PP and OO trajectories, a closer distance to PP does not inherently imply a greater distance from OO. To assess the relative distances in a multi-dimensional space, we introduced a trajectory similarity index (TSI). The TSI quantifies the differences in distances to PP and OO, normalized by the sum of those distances, such that a higher absolute TSI indicates greater similarity. The polarity of the TSI value reflects whether the activity is more similar to PP (positive) or OO (negative) (illustration in S9D Fig; see Materials and methods for details). Consistent with the analysis of distances to PP and OO activity, the TSI during the sample phase showed weak but significant directional activity in response to the given stimuli. As the delay phase progressed, however, the activity drifted toward the opposite direction (Fig 5H).

### Population activity of PPC neurons during the task

**Fig 6:**
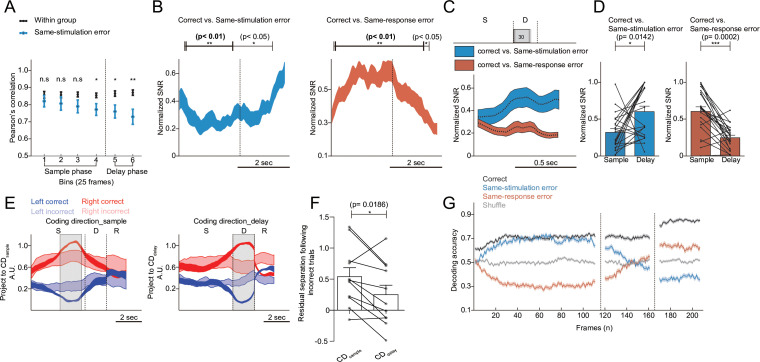
Population dynamics of posterior parietal cortex (PPC) neurons in correct and incorrect trials. **A.** Population activity correlations in the native space across time. Pearson’s correlation computed between trial-averaged population activity vectors within correct trials (black; within-group baseline) and between correct trials and same-stimulus error trials (blue). Data were pooled across 11 sessions (mean ± SEM). Correlations were calculated in six consecutive 25-frame bins (Bin 1–6), spanning the sample and delay phases. Significance was assessed using a nonparametric paired *t* test. *p*-values for each bin were as follows: Bin 1 (frames 21–45), *p* = 0.1475 (n.s.); Bin 2 (46–70), *p* = 0.1230 (n.s.); Bin 3 (71–95), *p* = 0.0537 (n.s.); Bin 4 (96–120), *p* = 0.0244 (*); Bin 5 (121–145), *p* = 0.0244 (*); Bin 6 (146–170), *p* = 0.0098 (**). **B.** Time-dependent changes in the normalized signal-to-noise ratio (SNR) to distinguish neural activity responses in correct and error trials using linear discriminant analysis (LDA). SNR was calculated using a sliding window of 50 frames, advancing one frame at a time. The vertical dashed line marks the initiation of the delay phase. Left Panel: mean normalized SNR between neural activity clusters for correct trials and same-stimulation error trials (e.g., LL vs. LR and RR vs. RL) as a function of time. Paired *t*-tests revealed significant differences: black horizontal line (frames 8–107, *p* < 0.05) and bold black line (frames 11–65, *p* < 0.01). Right Panel: mean normalized SNR between correct trials and same-response error trials (e.g., LL vs. RL and RR vs. LR). Significant differences are indicated by black line (frames 3–112, *p* < 0.05) and bold black line (frames 6–105, *p* < 0.01). Key: line thickness indicates mean ± SEM across sessions. **C.** Mean normalized SNR of LDA results during the delay phase. Blue Line: results comparing correct trials and same-stimulation error trials. Orange Line: results comparing correct trials and same-response error trials. Line thickness represents the standard error across sessions. **D.** Comparison of normalized SNR values during the sample phase (frames 66–115) and the delay phase (frames 121–170). Left Panel: correct vs. same-stimulation error trials. Right Panel: correct vs. same-response error trials. Statistical significance was determined using Wilcoxon signed-rank test, denoted as *p* < 0.05**, p* *< 0.01**,** and **p* *< 0.001. Error bars indicate mean ± SEM across sessions. **E.** Projections of population activity along two coding dimensions: CD_sample_ (coding dimension for the sample phase) and CD_delay_ (coding dimension for the delay phase). Projections are shown for each direction (red: right; blue: left) and response accuracy (dark: correct; light: incorrect). Line thickness represents mean ± SEM across sessions. **F.** Residual separation is defined as (Right incorrect − Left incorrect)/(Right correct − Left correct). (Wilcoxon signed-rank test, *p* < 0.05). **G.** Decoding accuracy of neural activity over time for correct trials (black), same-stimulus error trials (blue), and same-response error trials (orange), using a support vector machine (SVM). Line thickness indicates mean ± SEM across sessions. Underlying data and analysis code for this figure are available at DOI: https://doi.org/10.5281/zenodo.15872574.

The activity trajectories in the principal component dimension were computed using the combined activity of direction-selective neurons across sessions. To investigate how these dynamics relate to overall population activity, we applied a complementary analysis using all recorded neurons without dimensionality reduction or selectivity-based sorting. Specifically, we computed trial-by-trial Pearson’s correlations between population activity vectors in the native space using 25-frame windows (~0.8 s) across the trial (Fig 6A). This approach, inspired by Schoonover and colleagues [21], allowed us to assess representational drift directly in the full neural population. While within-condition correlations among correct trials remained high throughout the trial, correlations between error and correct trials were slightly reduced during the sample phase and showed a more pronounced decline during the delay phase, consistent with degradation of memory-related activity structure. Furthermore, to examine the effect of behavioral performance on activity stability, we grouped sessions based on performance level (above versus below 70%) and compared the correlation between correct and error trials over time. Higher-performing sessions exhibited slightly greater correlations during the sample phase and a more pronounced decline during the delay phase (S13 Fig), suggesting that error-related drift may be more dynamically expressed during higher-proficiency task execution.

To further quantify how this drift impacted stimulus encoding, we next applied a decoding-based analysis. Specifically, we used linear discriminant analysis (LDA) to compare population activity during error trials with that observed in correct trials, where the stimulus direction was either the same or opposite. This method provided a measure of the signal-to-noise ratio (SNR) of the encoded signal across the population, where the signal represents the distance between the two responses in the activity space, and the noise accounts for the variance within the responses (see Materials and methods). We measured the SNR changes in error trials within a moving window of 50 frames in the hyperplane that best discriminates between correct left- and right-trials (Fig 6B). Consistent with the trajectory distances of direction-selective neurons, the SNR between correct and same-stimulation error trials (correct left trials [LL] versus left stimulus-right response [LR], and correct right trials [RR] versus right stimulus-left response [RL]) initially decreased, reaching saturation around the middle of the sample period (~ 30 frames). As the frames from the delay period were included (after the 70th frame), the SNR increased (Fig 6B, left). In contrast, the SNR between correct and incorrect trials with the opposite stimulation (same-response error, LL versus RL, and RR versus LR) exhibited an opposite pattern: an initial increase and saturation over 30 frames during the sample period, followed by a subsequent decrease during the delay period (Fig 6B, right). The patterns of same-stimulation and same-response errors mirrored the TSI dynamics observed for these error types in the PCA-based trajectory distance analyses (Fig 5H). While grouping 50 frames across trials ensures separation between groups, this analysis obscures the SNR changes during the delay period. To better visualize the SNR changes, we replotted the measured SNR for every 30 frames within the delay period (Fig 6C). While same-stimulation errors became more differentiated during the delay phase, a decreasing SNR was observed in same-response errors. Across all frames, during same-stimulation errors, PPC activity was relatively similar in the sample phase but diverged more significantly during the delay phase. In contrast, in same-response errors, the activity differences driven by stimulus direction in the sample phase diminished during the delay phase (Fig 6D). These results underscore the role of activity drift toward the opposite direction in contributing to erroneous STM. The most straightforward interpretation of the drifting population activity toward the wrong direction in error trials is that it reflects a degradation or confusion of the retained sensory information. Alternatively, this activity could be interpreted as developing premotor activity related to an incorrect upcoming response. To distinguish between these possibilities, we compared population activity during error trials with two different delay durations: 1.5 s and 2 s (S11 Fig). If the observed drift originated from opposite-direction premotor activity, we would expect a temporal shift in the onset of drift between the two conditions—specifically, a delayed onset in the 2-s delay trials compared to the 1.5-s ones. However, we found that the onset timing of drift was not significantly different between the two delay conditions (S11 Fig), arguing against the interpretation that the drifting activity represents premotor planning.

While our analysis using PCA and LDA revealed activity differences between error and correct trials, the observed activity differences may not directly reflect the representation of the directional content of sensory inputs. To address this, we employed a dimensionality reduction approach to identify population activity modes that distinguish relevant features, particularly the signal corresponding to the direction of motion in the visual stimulus (coding direction or CD). To directly compare signal direction-dependent activity modes, we computed projections along a vector in the activity space of all recorded PPC neurons that maximally differentiate sensory direction in the sample phase (CD_sample_) and the delay phase (CD_delay_) (Fig 6E). To compare the separation of directional signals, we projected the error trial activities onto this vector. In comparison to correct trials, the directional sensory signals were weaker and less distinguishable between directions, particularly during the delay phase of error trials (Figs 6E and 6F). We then asked whether the activity along the CD_delay_ axis in individual trials covaries with the reaction time (RT) of the task. To test this, we projected the population activity onto the same CD_delay_ vector and computed the difference in projection during the final 200 ms of the delay period. However, we found no significant correlation between RT and this late-delay activity difference (S12 Fig), suggesting that trial-by-trial variability in motor initiation timing is not strongly reflected in the delay-period PPC activity. It should be noted, however, that the delay in our behavioral setup was imposed by the movement of the lick port, which limited the range of RT variation to a relatively narrow window (<250 ms).

Consequently, to assess how well trial-by-trial population activity predicted behavioral outcomes, we trained a support vector machine (SVM) model using PPC activity from correct trials. PPC activity from randomly selected 70% of correct trials in a session was labeled according to stimulus direction to train decoder, and the remaining trials were used to test prediction accuracy (Fig 6G). In correct trials, the prediction accuracy of the classifier significantly increased above the shuffled control level (gray line) shortly after sample phase and remained elevated until the end of the delay period (black line), indicating reliable encoding of stimulus direction. We next tested how the same decoder performed on error trials. When using the classifier trained on correct trials with the same-stimulus direction (blue line), the prediction accuracy showed a slight temporal delay but eventually reached a level comparable to correct trials. This suggests that stimulus identity was initially represented in PPC even during error trials. However, during the delay period of error trials, accuracy declined rapidly, dropping below the chance level near the end, indicating a drift in population activity toward the opposite-direction representation. To further distinguish sensory and motor contribution, prediction was made using a classifier trained on correct trials with the same-response direction (orange line). Prediction accuracy was below chance throughout the same phase, suggesting that early PPC activity did not reflect response planning. Notably, decoding accuracy gradually increased during the delay period, rising above chance only near its end.

To further examine whether task proficiency modulates stimulus encoding in the PPC, we grouped sessions by behavioral performance using a 70% accuracy threshold. We then repeated the decoding analysis separately for high- and low-performance sessions. We found that high-performance sessions (>70%) exhibited higher decoding accuracy throughout the sample and delay phases, whereas decoding accuracy was markedly reduced in low-performance sessions (≤70%) (S14 Fig). These results suggest that PPC population activity more reliably encodes stimulus information in well-trained animals, while reduced performance is associated with weaker or less stable sensory representations.

Taken together, our results demonstrate that during error trials, especially in the delay phase, population activity in the PPC shifts toward patterns typically observed for the opposite stimulus direction. This suggests that STM errors are associated with a reversal or drift in stimulus-specific encoding within the PPC.

## Discussion

### Distinguished STM representation from navigational and movement signals in PPC activity during STM

In the current study, we observed propagating neural activity that tracked the sensory information being maintained during STM task performance. Similar observations have previously been reported in STM-based virtual T-maze tasks [6]. However, subsequent studies have questioned the interpretation of these findings, suggesting that choice-dependent activity sequences could be predicted by heading direction and the animal’s position in the virtual corridor, potentially reflecting a navigational signal instead of STM processing [6,13]. This perspective aligns with earlier studies demonstrating that PPC neurons exhibit activity changes associated with locomotor actions, such as turning and forward motion, particularly when these movements are linked to spatial references [11,12,22,23]. Distinguishing between PPC activity related to sensory information maintenance and that influenced by navigational context is therefore critical for a more accurate understanding of PPC function in STM. Sensory cue-dependent go/no-go tasks also present interpretational challenges, as the observed activity may reflect preparatory signals related to impulsive licking behavior or its suppression, rather than sensory maintenance [6,14,20,24]. In our study, we reexamined PPC activity during an STM task that required retaining visual information and making directional choices based on equivalent licking behavior, but without incorporating a spatial component. Remarkably, we found similar activity patterns in PPC neurons, with direction-selective activity sequences that predicted sensory direction. These patterns were observed not only in the absence of navigational signals but also during the delay period when no ongoing stimulus was present. Furthermore, disruptions in PPC activity patterns during error trials were consistent with a drift toward an erroneous representation of the stimulus direction, further supporting the interpretation that PPC activity reflects an STM signal.

An alternative interpretation of the PPC activity is that it reflects developing premotor signals, or STM of planned response. Indeed, PPC activity has been shown to exhibit task-related selectivity—including greater responses in false alarm trials compared to correct rejections—which has been interpreted as reflecting premotor planning or a memory trace of intended action [20]. Although the lick ports were introduced only after the delay period to minimize overt motor preparation, it remains possible that well-trained animals began forming internal motor plans in anticipation of the response phase.

While we cannot exclude this possibility that delay-period activity includes components of motor preparation, several observations suggest that the activity more likely reflects sensory memory dynamics. Across all trials, including error trials, PPC activity during the sample period and early delay period was determined by the direction of the sensory stimulus (Figs 5 and 6). In error trials, however, this activity gradually drifted toward the pattern associated with the opposite direction (Figs 5H and 6B). One might argue that delay activity reflects a mixture of early sensory encoding and late-emerging motor preparation. In line with this, Goard and colleagues demonstrated that PPC activity encodes both stimulus identity and choice [14]. However, in our data, the onset of this drift was unaffected by changes in delay duration (S11 Fig), making it unlikely that the drift simply reflects premotor timing. Moreover, it is difficult to reconcile the emergence of an opposite-direction motor plan while the original sensory representation remains intact. Importantly, the same pattern of drift persisted even after orthogonalizing response-period activity (Fig 6E), and we found no significant correlation between RT and late-delay activity, as quantified by the distance of CD_delay_ to the decision boundary, further arguing against a motor origin.

We therefore propose that the observed drift reflects either the degradation or reinterpretation of a stimulus-specific memory trace within the PPC. Given that delay-period PPC activity unfolds as a temporal sequence, such misrepresentation may lead to the activation of an opposite-directional sequence, which ultimately gives rise to incorrect motor preparation. In this sense, the drift may contribute to—or transition into—premotor activity later in the delay, as the system prepares a behavioral output based on an internally corrupted memory. This interpretation aligns with prior studies suggesting that PPC activity reflects internal estimates or reinterpretations of sensory stimuli, rather than veridical representations of stimulus identity [9,25–27].

### Difference in population activity in error trials

We hired GLM to classify the neurons based on their activity patterns. This method proved robust even at the single-trial level, as validated by permutation-based Z-score analyses (S5 Fig). While most neurons classified as phase-selective showed consistent trial-level activity, we also observed that some exhibited relatively low trial-wise selectivity. Many of these were expected to be jointly selective for both task phase and stimulus direction. Because our task included both leftward and rightward trials within each phase, neurons with conjunctive phase–direction selectivity may show reduced or even suppressed activity in nonpreferred directional trials, even during their preferred phase. This likely contributed to the skewed Z-score distribution observed in our validation analysis. Rather than indicating inconsistency or classification error, such cases reflect multiplexed encoding of the task structure, a characteristic feature of associative cortical areas like the PPC.

A substantial proportion of PPC neurons exhibited significant directional preferences, with over 30% demonstrating exceptionally high selectivity (PI > 0.9, Figs 1F and 2). Most direction-selective neurons were tuned to specific phases, rather than maintaining sustained activity across both the sample and delay periods (Figs 1F and 2). This high directional selectivity, or high PI, may be facilitated by the wiring motifs in the PPC, where co-selective neurons form strong and more frequent synaptic connections while inhibiting anti-selective neurons [28]. In the PCA-driven activity subspace, which captured over 70% of the activity of selective neurons (S8 Fig), trajectories corresponding to two directional visual stimuli began diverging immediately after stimulus onset and continued to separate throughout the delay period (Fig 5). Surprisingly, this differentiation persisted and even increased during the delay period, despite the absence of ongoing sensory stimuli. This observation aligns with the significantly greater direction selectivity observed in delay neurons compared to sample neurons (Fig 2C). These findings support the hypothesis that the PPC primarily functions to maintain differentiated sensory inputs acquired during the sample period, rather than to detect and encode these signals. The latter function may instead rely on early sensory cortical processing [6,14,29]. The progressive increase in activity divergence during the delay phase may reflect processes aimed at optimizing visual information retention while minimizing activity variability. This could occur concurrently with premotor neuron preparation for invariant motor responses [30,31]. Further studies systematically comparing PPC activity under varying stimulus durations and complexities could clarify these possibilities.

Our complementary multivariate population analyses consistently revealed sustained population-level differences in activity in response to distinct stimuli during correct trials (Figs 5 and 6). Although this encoding pattern involves distributed activity across multiple neurons, it resembles the increased or decreased sustained firing rates observed in PPC neurons of primates [8,32] and rats performing STM tasks [9]. These findings suggest that population activity differences in the mouse PPC may represent a form of evidence quality. Differences in PPC activity between cue directions that emerge during the sample period appear to underpin stimulus encoding, while their maintenance during the delay period reflects STM retention. Consistent with this model, we observed weaker directional differentiation and poorer maintenance of population activity differences during error trials. These results suggest that STM errors are associated with suboptimal sensory encoding and a gradual weakening of this information throughout the task (Figs 5 and 6). However, despite reduced activity distinction and some neurons “misinterpreting” stimuli during the sample phase of error trials, population-level PPC activity still encoded the correct direction to a substantial extent (Fig 6G).

### Increasing opposite directional activity of PPC neurons in error trials

The origin of the reduced distance in activity space observed during error trials remains unclear. However, changes in the animal’s arousal state may contribute to this diminished encoding. Arousal levels vary continuously, and neuronal responses in sensory cortices, as well as higher-order cortical areas such as the PPC, are critically modulated by attentional states [6,33–38]. During error trials, insufficient arousal could lead to reduced PPC neuron activity in response to preferred visual stimuli, resulting in weaker separation in activity space (Figs 3D, 4G, and 4H).

Nevertheless, this explanation does not account for neurons with negative PI values—those that exhibit stronger firing to the opposite directional stimuli compared to the preferred direction. For these neurons, we propose that PPC activity encodes how the stimulus was ‘interpreted’ rather than its identity, particularly for directional delay neurons and, to a lesser extent, for sample neurons (Figs 3 and 4). This interpretation aligns with findings from macaque studies, where PPC neurons showed firing changes predictive of behavioral choices even when the stimulus contained 0% coherence in a random dot motion task [8].

Recent anatomical analyses have revealed that the probability and strength of connections among L2/3 PPC neurons depend on the similarity of their directional selectivity [23]. Furthermore, PPC subnetworks with opposing selectivity appear to suppress each other through mutual inhibitory connections [23,28,39,40]. Building on these findings and our results, we propose that direction-selective delay neurons form stronger subnetworks than sample neurons. In error trials, although the opposite-directional PPC activity during the sample phase does not initially alter the perceived direction of the stimuli, this activity may be amplified through the tightly interconnected delay neurons. This amplification could eventually result in an inversion of the encoded direction, leading to an error. This hypothesis is supported by our findings, which show that delay neurons exhibit significantly higher PI and RI values compared to sample neurons (Figs 2C and 4). Additionally, we observed a drift in population activity toward the opposite direction as the delay period progresses (Figs 5H and 6).

While our study focuses on the PPC, the role of PFC in working memory is also well-established, particularly in maintaining categorical, decision-related representations. Prior work has shown that PPC neurons encode sensory evidence in a graded manner, whereas PFC activity tends to reflect more discrete choice outcomes [8,9]. Our findings align with this distinction: we observed that during error trials, PPC population activity gradually drifted toward the representation of the opposite choice, particularly during the delay period. This slow drift, captured by trajectory analysis and reduced preference and reliability indices (PI and RI), supports the view that PPC maintains a continuous, sensory-based trace of stimulus identity—a structured memory buffer, encoding high-dimensional, sensory-informed signals that remain plastic over the course of the trial. In contrast, the PFC may operate downstream to read out these representations and commit to a choice. Thus, our results highlight the PPC as a locus of stimulus retention, in contrast to the PFC’s role in decision commitment, and suggest that STM errors may arise from corrupted maintenance of sensory information upstream of final motor decisions.

### Limitations of the study

Due to the limited number of task-relevant neurons recorded in a single trial and the inherent noise in cortical activity, much of our analysis was based on averaged activity across numerous trials of the STM task. Consequently, it was not feasible to reliably track the onset and amplification of inappropriate directional firing on a per-trial basis. As such, both the origin of erroneous activity within the subnetwork and the mechanisms underlying its amplification remain unresolved. Additionally, although PPC activity was recorded while mice were head-fixed to perform the task, unintended movements or fluctuations in arousal state may have occurred, potentially modulating PPC activity [33,36]. Future studies could explore [38,41] trial-to-trial variability under varying attentional conditions using high-density electrophysiological recordings, which may provide deeper insights into these dynamics [42–46]. In addition, while our findings emphasize drift-like dynamics in PPC activity during errors, the underlying cognitive content of these signals remains unclear. Specifically, it remains an open question whether the observed activity reflects memory of the sensory input identity (“the signal was left”), a planned action associated with that input (e.g., “I need to lick left when the port appears”), or a mixture of both. Finally, although we observed a significant relationship between task performance and neural drift when comparing high- and low-performance sessions, the broader role of training history—and how it shapes the stability and discriminability of population activity, particularly in representing sensory inputs—remains to be fully understood. Addressing these questions will be essential for refining our understanding of how the PPC supports STM and decision-making.

### Conclusions

In conclusion, our study advances the understanding of the neural mechanisms underlying sensory encoding and retention in STM tasks, highlighting the pivotal role of the PPC in these processes. By elucidating the relationship between PPC activity patterns and task performance, we provide valuable insights into the cognitive functions of the PPC, with significant implications for understanding STM errors. Given that impaired STM performance is a hallmark symptom of schizophrenia [47–49] and an endophenotype of other neuropsychiatric disorders [45], understanding [50] the nature of STM errors holds considerable translational relevance. Our findings pave the way for future research aimed at developing novel therapeutic approaches for these conditions. Moreover, exploring trial variability and attentional conditions with advanced recording techniques will deepen our understanding of PPC function in STM and its disruption in disease.

## Materials and methods

### Animals

All animal experiments were approved by the Animal Experiment Ethics Committee of the Korea Brain Research Institute (KBRI) (approval no. IACUC-17-00031). The experiments were performed using male C57BL/6J-Tg(Thy1-GCaMP6f)GP5.5Dkim/J transgenic mice (*n* = 6).

### Cranial window and head plate preparation

Mice aged 8–12 weeks underwent cranial window and head plate implantation surgery. The imaging window was centered over the PPC using stereotaxic coordinates (–2.0 mm AP, 1.75 mm ML), as defined by previous anatomical studies [16]. To functionally confirm the general location, we verified calcium responses to brief auditory stimuli with hand clap under low-magnification imaging, consistent with the multisensory responsiveness of the PPC. In a subset of animals (*n* = 4), we further validated the location by creating a small laser-induced lesion at the imaging site and confirmed the anatomical position post hoc via histological analysis (S1 Fig).

To alleviate pain and reduce inflammation, they received subcutaneous injections of ketoprofen (5 mg/kg) prior to surgery. Deep anesthesia was induced using a mixture of 1–2% isoflurane or a ketamine/xylazine solution (100 mg/kg and 10 mg/kg, respectively). Anesthesia was confirmed by the absence of the pedal withdrawal reflex. Both eyes were covered with petroleum jelly, and the incision site was shaved. The scalp was sterilized with ethanol and betadine before being excised. Using a scalpel, the periosteum was removed, and the skull surface was roughened with a drill burr (Miniature Carbide Burr Drill Bits Set, Stoelting) to ensure proper adhesion of the dental cement. The skull was rinsed with Ringer’s solution, and a craniotomy approximately 3 mm in diameter was performed over the PPC of the right hemisphere. The craniotomy was sealed with a double-layered glass coverslip (3/5 mm, Harvard Apparatus) combined with NOA71 adhesive (Norland) and secured with surgical adhesive (Vetbond, 3M). The dura mater remained intact. A tailor-made, commercially pure titanium head plate (rectangular, 19 mm × 12 mm × 1 mm) with a 4.5 mm central opening was affixed on top of the window using dental cement (Super-Bond C&B kits, Sun Medical, darkened with black pigment). After surgery, mice were placed in a warmed chamber for at least 4 h for recovery and post-operative monitoring. Subsequently, the animals were housed individually in cages.

### Behavior task

After full recovery from surgery, the mice were provided with limited water (1 mL/day), and their body weights were monitored regularly. If a mouse’s body weight dropped below 75% of its initial weight, water restriction and training were halted. The mice were trained to perform a delayed match-to-sample task while their heads were fixed in place using a holder (modified MAG-1, Narishige Scientific Instrument, Tokyo, Japan). The visual signal for discrimination consisted of high-contrast sine-squared wave gratings moving either left/right or down/right, with specific spatial (3 cm/cycle) and temporal (2 Hz) frequencies. This signal was displayed on a monitor positioned directly in front of the mice at a distance of 25 cm. Custom-written software running on a Raspberry Pi 3 (RPi3) controlled both the stimuli generation and behavioral training. Mice were trained separately in either the left/right or down/right directions, and the data from both sets of trials were pooled and analyzed together.

The position of the drinking spouts was individually adjusted for each mouse. The lick detection circuit and reward solenoid were controlled by an Arduino Mega2560 (rev 3, Arduino), which was connected to the Raspberry Pi 3 (RPi3). During training, the mice earned the majority of their daily water intake (1 mL) through task performance. Each visual stimulus lasted for 4 s, followed by a 2-s pause. After this pause, the spouts quickly moved to a position accessible by the mouse’s tongue for 1 s. A successful lick (i.e., licking the left-hand spout in response to a leftward or downward-moving signal, or licking the right-hand spout in response to a rightward-moving signal) during this window resulted in a reward of approximately 5 µL of tap water. Incorrect licks were not rewarded and were followed by a 1.5–2-s time-out, after which the trial continued. After the reward period or an incorrect lick, the spout retracted quickly and stayed out of reach until the next trial, which began 4 or more seconds later. Training progressed in stages: Initially, the mice learned to lick a nonmoving central lick port in response to either visual cue. Next, they were trained to match the visual direction with the corresponding reward spout. Subsequently, the delay was gradually increased by introducing moving lick ports. Once the mice consistently performed with over 70% accuracy at a 1.5- or 2-s delay, both behavioral performance and PPC activity were recorded for further analysis.

### In vivo two-photon calcium imaging

We performed two-photon imaging using a HyperScope (Scientifica) and ScanImage (MBF Bioscience) setup, powered by a Ti:Sapphire laser (Chameleon Ultra II, Coherent). The imaging system was configured to target the GCaMP6f calcium indicator, which was excited at 910 nm using a 16× water immersion objective lens (N16XLWD-PF, Nikon). A custom-made light barrier was attached to the objective lens to isolate the imaging system from any visual stimuli. Images were captured at a resolution of 512 × 512 pixels (700 µm × 700 µm region of interest [ROI]) at a frame rate of 30 Hz in layer 2/3 of the PPC. The average laser power at the objective lens was kept below 100 mW to minimize tissue damage. The imaging and behavioral systems were synchronized via a frame trigger signal output, which was counted by an Arduino Mega 2560 (rev 3).

### Image preprocessing and cell selection

Calcium imaging data were processed using the CaImAn software framework. Motion correction was applied in segments of 128 × 128 pixels, with each segment overlapping by 48 pixels to ensure continuity across frames. For neuronal source identification, the ‘greedy_roi’ method was employed, with expected neuron sizes set to 4 × 4 pixels for detailed imaging and 3 × 3 pixels for full-brain imaging. Only components with a signal-to-noise ratio (SNR) of at least 2.0 and 90% pixel correlation were considered reliable. Fluorescence signal decay rates were normalized by applying Z-scoring. A decay rate of 3.0 s was used for indicators localized in the nucleus, and 1.0 s for those in the cytosol. To align neural activity with behavioral actions, all time series data were synchronized to a uniform sampling rate of 30 Hz, corresponding to the frame capture times of the camera and microscope.

### Automated event-related neuron detection in calcium imaging data

We used a GLM to regress recorded calcium signals against a time series of task events. We propose a novel method for automatically searching event-related neurons in calcium imaging data (S15 Fig). Our method searches the ROI pixelwise using event timing information. By utilizing a statistical method to determine the relationship between the calcium trace of each pixel and the event timing, the searched ROI pixels are clustered. This process enables the automatic detection of event-related ROIs without requiring any additional detection procedures while also enabling the detection of components with low SNR or fine size as ROI.

The proposed method determines the event relevance of calcium traces to detect event-related neurons in calcium imaging data. Rather than relying on the morphological shape of the neuron for ROI detection, the proposed method determines the event relevance of each pixel’s calcium trace independently. If ROI detection is performed by reflecting the neuron’s morphological shape, the detection performance may decrease with low SNR or fine-size components.

We computed the mean Δ*F*/*F* for the GLM-defined preferred and nonpreferred phases on a trial-by-trial basis. A selectivity index was defined as the difference between these values per-trial. To generate a null distribution, we shuffled phase labels across trials 1,000 times and recalculated the selectivity index. Neurons were considered significantly selective if their observed index exceeded the 95th percentile of the null distribution.

Event timing refers to the points in time when tasks or stimuli occur, and the calcium signal of event-related neurons should show a clear response at these times. In contrast, nonevent timing refers to periods of spontaneous activity that are unrelated to the event. To categorize neurons, we constructed a GLM using event-related temporal templates aligned to the timing of each task event. Specifically, the key task events defined on correct trials were: (1) right- and left-signal sample phases (signal onset to frame 120), (2) right- and left-signal delay phases (frame 121–170 after signal onset), and (3) right- and left-signal reward phases (lick port touch onset to 45 frames afterward). These temporal templates were used as binary regressors indicating the presence of each event over time, aligned to trial timing. S15 Fig illustrates this classification procedure and provides visual examples of neurons selected by the model.

Neurons associated with events exhibit strong activation at event timing and minimal activation at nonevent times. Therefore, we define a spike train $\tilde{s}(t)$ of the calcium signal most strongly associated with the event:


$$\tilde{s}(t)=\sum_{i=\text{1}}^{k}\beta_{i}\delta (t-t_{i})$$ (1)

where $\tilde{s}(t)$ is composed of the product of the β (amplitude of the spike) and the impulse train. An event-related neuron is expected to activate at least once after an event. Denoting the *i*^*th*^ event timing by $t_{i}$, $\tilde{s}(t)$ becomes a spike train of event-related neurons with an amplitude of $\beta_{i}$ at event timings.

The temporal component of calcium dynamics is determined by the activity of a calcium indicator. When the calcium indicator is combined with calcium, luminous intensity changes. The fluorescence trace of the calcium indicator was modeled in the form of a double exponential and is expressed as:


$$c(t)=e^{\frac{-t}{\tau_{d}}}-e^{\frac{-t}{\tau_{r}}}$$ (2)

where $\tau_{r}\;$and $\tau_{d}$ are the time constants for the increase and decrease of fluorescence, respectively [51].

We express the modeled event-related calcium trace $\tilde{y}(t$of the neuron by the fluorescence trace model of the calcium indicator c(t) with the convolution of the spike train $\tilde{s}(t)$.


$$\tilde{y}(t)=\int_{-\infty }^{\infty }\tilde{s}(t-\tau )c(\tau )\text{\textbackslash{} }d\tau$$ (3)

We defined the event-related calcium trace model $\tilde{y}(t)$. The ROI was detected by determining the relationship between this model and the pixel’s calcium trace y(t) using the least square method and 1-sampled *t* test. Through the least square method, it is possible to find *t*he estimator of the regression coefficient β (the amplitude of the spike in eq (1)) that minimizes the residual of the pixel’s calcium trace y(t) and the event-related calcium trace model $\tilde{y}(t)$ of the neuron. The β estimator matrix $\hat{\beta }\;$up to the kth event was expressed as:


$$\hat{\beta }=\left[\beta_{\text{1}}\beta_{\text{2}\;}{\text{\textbackslash{} }\beta }_{\text{3}}\cdots \beta_{k}\right]={\left\|y(t)-\tilde{y}(t)\right\|}^{\text{2}}$$ (4)

Notice that $\tilde{y}(t)$ has a weight c(t) for event timing. By c(t), as the event timing and activation timing are closer, the value of the regression coefficient β increases. It was determined whether a neuron is event-related through the distribution of β. As the mean of $\hat{\beta }$ is close to 0 or the variance of $\hat{\beta }$ is larger, the activation timing of neurons has little relation to the event timing. Therefore, we tested the null hypothesis that *β* is extracted from a normal distribution with a zero mean and unknown variance, using a 1-sampled *t* test. At this time, the test statistic is as shown:


$$test\;statistic=\frac{\overset{―}{\beta }}{\frac{s}{\sqrt{k}}}\mathrm{\backslash ]}$$ (5)

where $\underset{―}{\beta }\;$ is the mean of the $\hat{\beta }$, s is the standard deviation of the $\hat{\beta }$, and k is the size of $\hat{\beta }$, also known as the trial number of events. The proposed method uses a *p*-value of 0.05 to determine a pixel with a statistically significant difference from a pixel of an event-related neuron.

When the event association determination is completed for all pixels of the calcium image, the proposed method finally defines the event-related ROI by clustering the pixels associated with the event. The proposed method clusters pixels to ROIs automatically, where the clustering is performed based on the Moore-Neighbor tracking algorithm and determines connectivity between pixels [52]. Finally, the proposed method provides ROI clusters corresponding to event-related neurons (S15 Fig). In a subset of sessions, we observed gradual drifts in overall neuronal activation over time, likely due to slow baseline shifts or signal drift. To prevent such nonstationarities from contaminating the analysis of incorrect-trial activity, we implemented an additional exclusion criterion. Although the neurons included in this analysis were identified as phase- and direction-selective using a GLM, we excluded any neuron that exhibited higher mean activation in response to the nonpreferred (opposite) signal compared to the preferred signal during its selective phase.

### Selectivity

To identify neurons selectively active during specific behavioral phases and conditions, we employed a uniquely developed GLM followed by ROC-based indices to quantify selectivity and reliability. These indices were calculated based on Δ*F*/*F* values extracted from specific behavioral epochs (sample, delay, or reward periods) as defined by the task structure.

#### Preference Index (PI).

The PI quantifies the directional selectivity (left versus right trials) of a neuron during a given phase. For each neuron, we computed the mean Δ*F*/*F* during the selected phase (e.g., sample or delay) for all left and right trials separately. We then used these trial-averaged values to generate two distributions and applied receiver operating characteristic (ROC) analysis to evaluate how well an ideal observer could distinguish between them. The PI was then calculated as:


2×(auROC−0.5) (6)

This yields a value ranging from −1 (strong left preference) to +1 (strong right preference), with 0 indicating no directional bias.

#### Reliability Index (RI).

While the PI assesses average differences, the RI evaluates trial-to-trial consistency of a neuron’s direction selectivity. For this, we took the Δ*F*/*F* values from each individual trial, without averaging, during the phase of interest. These single-trial values were grouped by trial type (left versus right) and submitted to the same ROC analysis as above, yielding the RI, using the same formula as the PI.

#### Phase selective index (PSI).

PSI is derived by comparing the trial-averaged neural activity during the phase of interest with that from nonrelated phases. By using these indices, we were able to quantitatively assess the degree to which individual neurons preferentially respond to different trial types, offering insights into their roles within the observed behavioral patterns.

#### Trajectory Similarity Index (TSI).

To quantify the evolving similarity of trial-wise population activity to prototypical correct-trial trajectories, we computed a Trajectory Similarity Index (TSI). For each trial, we calculated its Euclidean distance in PCA space to the PP (preferred direction correct) and OO (opposite direction correct) trajectories at each time bin. The TSI was then defined as


$$\text{TSI}\;=\;{(d}_{\text{OO}}\;-d_{\text{PP}})\;/\;{(d}_{\text{OO}}+\;d_{\text{PP}})$$ (7)

where $d_{\text{PP}}$ and $d_{\text{OO}}$ are the distances from the trial to the PP and OO trajectories, respectively. This normalization bounds the index between −1 and +1, with positive values indicating greater similarity to PP and negative values indicating greater similarity to OO.

#### PCA.

We used Principal Component Analysis (PCA) on neurons sorted using the GLM. The goal was to infer an intrinsic low-dimensional manifold representing the collective dynamics of task-selective neurons. For this analysis, we focused on correct trials only. The data used for PCA consisted of trial-averaged calcium activity. For each neuron, the fluorescence signal was averaged across all correct trials of its preferred stimulus direction. This resulted in a data matrix *X* of size *M* × *N*, where *M* is the number of imaging frames per-trial and *N* is the number of neurons included in that session (*N* = *N*__sample_ + *N*__delay_ + *N*__reward_, as defined by GLM classification). To account for the fact that direction-selective neurons may respond preferentially to leftward or rightward stimuli, we aligned all neurons to their preferred direction prior to PCA. Specifically, for neurons that preferred left stimuli, we flipped the trial labels and time-series to match the right-preferring format, so that population responses could be interpreted in a common aligned space. This alignment allowed us to pool directional neurons for dimensionality reduction. To ensure comparability across neurons with different baseline activities or amplitudes, we applied Z-score normalization to each column of the matrix *X*, such that each neuron’s activity vector had a mean of 0 and standard deviation of 1. PCA was then performed on the resulting normalized data. We found that the first five principal components explained a substantial portion of the variance across correct trials (e.g., 72.4% for right-preferring neurons, 69.0% for left-preferring neurons, and 70.4% for all directional neurons combined).


$$Z_{\text{correct}}=\frac{\left(X-\mu \right)}{\sigma }$$ (8)

where μ is the mean and σ is the standard deviation of neuron activity.

We calculated the covariance matrix, C, from the normalization data Z, to understand how the variables relate to one another.


$$C_{\text{correct}}=\frac{1}{N-1}Z_{\text{correct}}^{T}Z_{\text{correct}}$$ (9)

where N is the number of neurons.

To further analyze the neuronal activity data, we computed the eigenvalues and eigenvectors of the covariance matrix C. The eigenvectors correspond to the directions of maximum variance in the data, while the eigenvalues indicate the magnitude of these variances. Neuronal activity data were projected onto the principal components to transform the data into a new space defined by these components. To compute relative changes in incorrect trials compared to correct trials, we projected the incorrect data onto the first five principal components $(V_{𝐜𝐨𝐫𝐫𝐞𝐜𝐭}$) derived from correct trials, resulting in a reduced-dimensional representation $Z_{𝐩𝐜𝐚}$ (10). These five components captured the majority of population-level variance in correct-trial activity (>70%).


$$Z_{\text{pca}}=Z_{\text{incorrect}}\;\times V_{\text{correct}}$$ (10)

where $V_{\text{correct}}$ represents eigenvectors and $Z_{\text{pca}}$ represents the data transformed into the principal component space.

To assess whether population activity in error trials deviated from the structure observed in correct trials, we performed a split-trial PCA analysis. For each session, PCA was computed on activity from all selective neurons, using calcium signals averaged across 50% of correct trials (randomly chosen). The remaining correct trials and all error trials were then projected into the resulting principal component space. This procedure allowed us to compare population dynamics without circularity.

### Coding direction

This procedure closely follows previously described methodologies [53,54]. The activity of n neurons, recorded simultaneously during the entire session, was analyzed (Fig 6E). The population activity of n neurons formed a trajectory in the n-dimensional activity space during each session, with each dimension representing the activity of a single neuron. In this activity space, we identified directions (or modes) that maximally separated the neuronal pathways associated with different trial conditions and specific phases. For trials with lick-correct left and lick-correct right instructions, the trial-averaged activity differences of the n neurons were computed within a 1.7-s window (frames 121–170) following the visual cue offset. This resulted in the delay mode (${\text{CD}}_{\text{delay}}$), which was calculated as follows:


$$\Delta \underset{―}{r}={\underset{―}{r}}_{\text{correct}\;\text{right}}\;-\;{\underset{―}{r}}_{\text{correct}\;\text{left}}$$ (11)

The resulting vector was normalized by its $l^{2}$ norm, yielding a weight vector, with one weight assigned to each neuron i, as follows:


$$m=\frac{\Delta \overset{―}{r}}{\sqrt{\sum_{i}^{N}{\left|\Delta \overset{―}{r_{i}}\right|}^{\text{2}}}}$$ (12)

Projections of the neural activity onto the mode over time were computed as follows:


$$P^{k}={{(X}^{k})}^{T}m$$ (13)

where $X^{k}$ is the N×T matrix of averaged activity of neurons over time for session k.

To maintain consistency in the analysis despite the varying number of neurons recorded in each trial, we normalized each mode by its $l^{2}$ norm, as indicated in eq (12). This normalization ensured that the projected neural activity, $P^{k}$ remained unaffected by the quantity of neurons recorded simultaneously. In instances where a specific neuron did not show any specific activity during a particular trial, we assigned a weight of zero to that neuron in both eqs (12) and (13), for calculating the projected activity for that trial. This approach guaranteed that the analysis could accurately reflect the contribution of active neurons in each trial, without skewing results due to differences in neuron sampling across trials.

The sample mode (${\text{CD}}_{\text{sample}})$and the reward mode (${\text{CD}}_{\text{reward}})$ were defined to capture distinct aspects of neural activity corresponding to different phases of the trials, specifically focusing on the behavioral outcomes of licking correctly to the right or left.

${\text{CD}}_{\text{sample}}$: This mode was determined by constructing an n×1 vector representing the differences in the average activity of n neurons during trials that resulted in correct licks to the right or left. These differences were averaged over a 1.7-s window (frames 66–115) coinciding with the beginning of the stimulus presentation. By focusing on this early window, ${\text{CD}}_{\text{sample}}$ captures the neural activity patterns triggered by the initial stimulus, reflecting how neurons differentiate between trial types in response to the stimulus.

${\text{CD}}_{\text{reward}}$: similarly, this mode was defined by an n×1 vector, but it focused on the average activity differences of n neurons during trials with correct licks to the right or left, within a 1-s window (from frame 171 to frame 200) at the conclusion of the delay phase. The ${\text{CD}}_{\text{reward}}$ specifically targets the neural response patterns associated with the anticipation or receipt of a reward, highlighting how neuronal activity aligns with behavioral outcomes following the stimulus-response interval.

In addition to the selective modes that focused on differentiating neural activity based on specific trial outcomes, we identified a nonselective ramping mode (${\text{D}}_{\text{reward}}$). This mode was designed to capture general changes in neural activity towards the end of the delay phase, irrespective of the trial outcome (whether the lick was correct to the right or left). To compute this ramping mode, we adopted a methodology similar to that used for the reward mode. However, instead of distinguishing between trial outcomes, we aggregated neural responses from both correct-right and correct-left licks. We then calculated the difference in trial-averaged activity during the last second of the delay phase (from frame 141 to frame 170) compared to the first second of the reward phase (frames 171–200).


$$\Delta \underset{―}{r}={\underset{―}{r}}_{\text{correct}\;\text{reward}}\;-\;{\underset{―}{r}}_{\text{correct}\;\text{last}\;\text{delay}}$$ (14)

To facilitate a direct comparison between the sample and delay phases while considering their distinct neural dynamics, we employed the Gram-Schmidt process for orthogonalization of these phases in a specific sequence [55]. This process was applied to the modes in two different orders: first, starting with the ${\text{CD}}_{\text{reward}}$, then the ${\text{D}}_{\text{reward}}$, followed by the ${\text{CD}}_{\text{sample}}$; and second, beginning with the ${\text{CD}}_{\text{reward}}$, then the ${\text{D}}_{\text{reward}}$, and finally, the ${\text{CD}}_{\text{delay}}$. Orthogonalization using the Gram-Schmidt process ensures that each mode represents a unique aspect of neural activity, independent of the others, thus allowing for a clearer analysis of the distinct contributions of sample and delay phases to overall neural dynamics.

Furthermore, to assess the neural activity’s alignment with behavioral outcomes—specifically, the differences between correct and incorrect responses—we projected the trial-averaged activity from incorrect trials onto the modes defined by correct trials across sessions. This projection allowed us to compare the neural activity associated with correct and incorrect trials within the same mode, providing insights into how specific patterns of neural dynamics correlate with behavioral performance. This approach helped in understanding the neural underpinnings of decision-making and error processing by quantifying the extent to which neural activity during incorrect trials deviates from the established patterns of correct trials.

After projecting the neural activity data along the direction defined by CD (which represents the difference in neural activity between conditions), we normalized the resulting projections. This normalization involved adjusting the projections based on the average value obtained from correct rightward trials during the specific phase associated with each mode, for every session. This step ensured that the comparison across different trials and sessions was on a consistent scale, facilitating a more accurate analysis of the neural activity patterns.

To further investigate the differences in neural activity patterns between incorrect and correct trials, we introduced the concept of “residual separation.” This metric quantifies the deviation in the neural trajectories of incorrect trials from those of correct trials. The residual separation effectively captures the extent to which the neural representation of incorrect trials diverges from the expected pattern observed in correct trials, providing insights into the neural mechanisms underlying error processing and decision-making accuracy. This analysis allowed us to better understand how the brain’s neural activity aligns with behavioral outcomes and how it adapts or fails in cases of incorrect responses.


$$\text{Residual}\;\text{separation}=\frac{{\text{mean}(\text{Traj}}_{\text{incorrect}\;\text{right}})\;-\;\;\text{mean}({\text{Traj}}_{\text{incorrect}\;\text{left}})}{{\text{mean}(\text{Traj}}_{\text{correct}\;\text{right}})\;-\;\text{mean}({\text{Traj}}_{\text{correct}\;\text{left}})}$$ (15)

Mean (Traj) represents the averaged trajectory at the time corresponding to each mode.

### Linear discriminant analysis

This procedure closely follows the previously described methodologies [56,57]. In investigating a neural network that processes stimulus-tuned input under noisy conditions, with the objective of accurately identifying the stimulus, we examined the characteristics of neural signals. Neural signals are assumed to be modeled as a combination of stimulus-driven signals and noise. The noise is characterized as temporally uncorrelated and independent of the stimulus. The analysis identifies a statistically optimal strategy for distinguishing between two different signals. This strategy involves performing a linear projection combined with temporal filtering of the input time series. By applying this approach, we derived the optimal projection weights and filtering mechanisms. Additionally, we calculated the signal-to-noise ratio (SNR) achieved through specific combinations of projection and filtering. The trials were categorized into four groups based on the mouse’s response to directional cues: (1) Trials where the mouse licked right in response to a right-directed cue. (2) Trials where the mouse licked left in response to a left-directed cue. (3) Trials where the mouse licked left in response to a right-directed cue. (4) Trials where the mouse licked right in response to a left-directed cue.

For the task of distinguishing between two types of signals (as an example, only one pair of response and cue listed above)$\;S_{i}$, where i=1 corresponds to a correct right decision and i=2 to a correct left decision, we consider the dynamics of signals at each time step t. The assumption of input signal vector, $u\left(s_{i},t\right)$, follows a multivariate normal distribution, characterized by a stimulus-dependent mean and stimulus-independent covariances. The mean signal vector for the stimulation $S_{i}$ is denoted as $m_{i}$ and total mean vector as m. The within signal class covariance matrix is calculated as:


$$S_{W}=\;\sum_{i=\text{1}}^{\text{2}}\sum_{t\in S_{i}}(u\left(S_{i},\;t\right)-\;m_{i}){\left(u\left(S_{i},\;t\right)-\;m_{i}\right)}^{T}$$ (16)

The covariance between signal class matrix is calculated as:


$$S_{B}=\;\sum_{i=\text{1}}^{\text{2}}(m_{i}-m){\left(m_{i}-m\right)}^{T}.$$ (17)

From these matrices, transformation matrix of LDA can be derived


$$W=\;{S_{W}}^{-\text{1}}S_{B}.$$ (18)

The eigenvector $v_{\text{MAX}}$ of maximal eigenvalue $\lambda_{\text{MAX}}$ is selected as a maximum separation vector for discrimination of signal that we can project signal to calculate the SNR.


$$y\left(S_{i},\;t\right)=\;{v_{\text{MAX}}}^{T}u\left(S_{i},\;t\right)$$ (19)

$${\text{SNR}}_{\text{12}}(t)=\;\frac{\sum_{t^{\prime }=t}^{t+w}\left|y\left(S_{\text{1}},\;t\prime \right)-\;y\left(S_{\text{2}},\;t\prime \right)\right|}{w\sqrt{\frac{\text{1}}{\text{2}T}\sum_{t^{\prime }=\text{0}}^{all,T}\sum_{i=\text{1}}^{\text{2}}{{(v}_{\text{MAX}}}^{T}(u\left(S_{i},\;t\prime \right)-m_{i}{))}^{\text{2}}}}$$ (20)

To standardize comparisons across different sessions and ensure a uniform basis for analysis, the SNR values observed were normalized against the maximum SNR value observed in the respective session. This step was critical for drawing direct comparisons across sessions, effectively illuminating the signal quality differences between them. The utilization of Matlab for all data analysis provided a robust framework for dissecting and understanding the dynamic processes underlying the behavioral responses.

## Supporting information

S1 Fig Localization of two-photon calcium-imaging field of view. Representative coronal brain sections showing the location of the two-photon calcium-imaging site. A focal lesion was made on the cortical surface using an 800 nm laser to mark the imaging site. The position was subsequently verified by co-registering the histological slices to the Allen Mouse Brain Atlas using QuickNII software. Underlying data and analysis code for this figure are available at DOI: https://doi.org/10.5281/zenodo.15872574.
(TIFF)

S2 Fig PPC dynamics along the coding direction in the STM task, analyzed separately for sorted and unsorted neurons. **A.** Pearson’s correlation of activity vectors computed from sorted PPC neurons using 0.5-second bins, aggregated across 11 sessions (6 mice, 2,437 neurons). **B.** Pearson’s correlation of activity vectors from unsorted PPC neurons using the same parameters (11 sessions, 6 mice, 2,304 neurons). Underlying data and analysis code for this figure are available at DOI: https://doi.org/10.5281/zenodo.15872574.
(TIFF)

S3 Fig The proportion of directional and nondirectional neurons identified by a GLM. **A.** The fraction of sample-phase specific neurons showing significant fluorescence changes in response to left, right, or both directional visual stimulations. **B.** The same fractions for delay-phase specific neurons. **C.** The same fractions for reward-phase neurons. Underlying data and analysis code for this figure are available at DOI: https://doi.org/10.5281/zenodo.15872574.
(TIFF)

S4 Fig Reliability index of neurons, categorized into phase-specific directional and nondirectional groups. **A.** Histogram of reliability indices (RI) for GLM-defined all directional single-phase specific neurons in correct trials (light blue), and shuffled trials (gray). **B.** Histogram of RI for GLM-defined all nondirectional single-phase specific neurons in correct trials (orange), and shuffled trials (gray). **C.** Cumulative distribution function (CDF) of variable for directional single-phase specific neurons (light blue line) and nondirectional single-phase specific neurons (orange line). The distributions of RI values for directional and nondirectional neurons were significantly different (two-sample Kolmogorov–Smirnov test, *D* = 0.379, *p* < 0.001). **D.** Histogram of RI for GLM-defined directional sample-phase specific neurons in correct trials (light blue), and shuffled trials (gray). **E.** Histogram of RI for GLM-defined nondirectional sample-phase specific neurons in correct trials (orange), and shuffled trials (gray). **F.** CDF of variable for directional sample-phase specific neurons (light blue line) and nondirectional sample-phase specific neurons (orange line). Among sample phase-selective neurons, the distributions of RI values for directional and nondirectional neurons were significantly different (two-sample Kolmogorov–Smirnov test, *D* = 0.369, *p* < 0.001). **G.** Histogram of RI for GLM-defined directional delay-phase specific neurons in correct trials (light blue), and shuffled trials (gray). **H.** Histogram of RI for GLM-defined nondirectional delay-phase specific neurons in correct trials (orange), and shuffled trials (gray). **I.** CDF of variable for directional delay-phase specific neurons (light blue line) and nondirectional delay -phase specific neurons (orange line). Among delay phase-selective neurons, the distributions of RI values for directional and nondirectional neurons were significantly different (two-sample Kolmogorov–Smirnov test, *D* = 0.378, *p* < 0.001). **J.** Schematic illustration of PI calculation. Trial-aligned calcium activity from correct rightward and leftward trials is averaged across trials within each task phase: sample (black), delay (blue), and reward (tan). For PI calculation, activity during the delay phase (highlighted by the dotted oval) is compared between right and left trials using receiver operating characteristic (ROC) analysis. PI is defined as (auROC − 0.5) × 2, providing a normalized measure of directional selectivity. Bottom left: ROC curve from an example delay-selective neuron preferring rightward trials. Bottom right: normalized Δ*F*/*F* distributions from right (red) and left (blue) trials for the same neuron. **K.** Schematic illustration of Reliability Index (RI) calculation. Trial-aligned Δ*F*/*F* activity during the delay phase is extracted from all the correct rightward and leftward trials. Signals are normalized to each neuron’s maximum Δ*F*/*F* across the entire trial and pooled separately for each condition. These distributions are compared using receiver operating characteristic (ROC) analysis. RI is defined as (auROC − 0.5) × 2, providing a normalized index of directional selectivity based on all frames during the delay period. Bottom left: example ROC curve for a right-directional, delay-phase selective neuron. Bottom right: histogram of normalized Δ*F*/*F* across all frames, comparing right (red) and left (blue) trials. Underlying data and analysis code for this figure are available at DOI: https://doi.org/10.5281/zenodo.15872574.
(TIFF)

S5 Fig Phase selectivity of GLM-defined Single-phase specific neurons compared between sorted and unsorted neurons in correct trials. **A.** Left Panel: histogram of phase selectivity indices (PSI) for GLM-defined sample-phase specific neurons in correct trials (light blue), and unsorted neurons in correct trials (gray). A more positive phase selectivity index indicates a more specific response to the corresponding phase. Middle Panel: histogram of PSI for GLM-defined delay-phase specific neurons in correct trials (light blue), and unsorted neurons in correct trials (gray). Right Panel: histogram of PSI for GLM-defined reward-phase specific neurons in correct trials (light blue), and unsorted neurons in correct trials (gray). **B.** Permutation-based validation of epoch-specific neuron sorting. To assess the accuracy of our classification, we computed each neuron’s mean activity on every trial during the Sample, Delay, and Reward epochs, then generated null Gaussian distributions for each epoch by randomly shuffling trial labels 1,000 times. For each epoch, we calculated the percentage of trials in which the neuron’s observed mean activity exceeded the one-tailed *p *< 0.05 threshold of the corresponding null distribution—an x-axis value of 100% therefore indicates that the neuron showed statistically significant, epoch-specific activity on every trial. **C.** Using the same permutation procedure as in Fig 5B, null Gaussian distributions were generated for each epoch. For each neuron, we then calculated its single mean activity across all trials in the epoch of interest and expressed its position within the corresponding null distribution as a Z-score. Higher Z-score values therefore, indicate stronger epoch-specific modulation of the neuron’s activity. **D.** Schematic illustration of Phase Selectivity Index (PSI) calculation for a task-phase–selective neuron. Neuronal activity from correct rightward and leftward trials is pooled and aligned to each task phase: sample (black), delay (blue), and reward (tan). To quantify phase selectivity, normalized Δ*F*/*F* signals during the phase of interest (e.g., the delay phase in this example) are compared to those from the other two phases (sample and reward) using receiver operating characteristic (ROC) analysis. PSI is defined as (auROC − 0.5) × 2, providing a normalized measure of delay-phase selectivity. Underlying data and analysis code for this figure are available at DOI: https://doi.org/10.5281/zenodo.15872574.
(TIFF)

S6 Fig Anatomical distances and functional similarities. **A**. The relationship between within-group and across-group distances for phase-selective neurons. **B.** The within-group and across-group distances for directional and non-directional neurons. Underlying data and analysis code for this figure are available at DOI: https://doi.org/10.5281/zenodo.15872574.
(TIFF)

S7 Fig Reliability index of the direction- and phase-selective neurons in error trials. **A.** Color-coded trial average Δ*F*/*F* of nondirectional single-phase selective neurons in error trials. Δ*F*/*F* was normalized to the maximum Δ*F*/*F* in correct trials and aligned based on the time-to-peak of each neuron. **B.** Histogram of PI of GLM-defined nondirectional single-phase selective neurons in correct (orange), error (red open), and shuffled (gray) trials. **C.** Histogram of PI of GLM-defined nondirectional sample-phase selective neurons in correct (black), error (red open), and shuffled (gray) trials. **D.** Histogram of PI of GLM-defined nondirectional delay-phase selective neurons in correct (blue), error (red open), and shuffled (gray) trials. **E.** Left Panel: Histogram of RI of GLM-defined nondirectional single-phase selective neurons in correct (orange), error (red open), and shuffled (gray) trials. Right Panel: CDF of the left histogram. Among nondirectional neurons, the distributions of RI values for correct and error trials were significantly different (two-sample Kolmogorov–Smirnov test, *D* = 0.132, *p* < 0.001), albeit with a smaller effect size compared to directional neurons. **F.** Left Panel: Histogram of RI of GLM-defined nondirectional sample-phase selective neurons in correct (orange), error (red open), and shuffled (gray) trials. Right Panel: CDF of the left histogram. Among nondirectional, sample phase-selective neurons, the distributions of RI values for correct and error trials were significantly different (two-sample Kolmogorov–Smirnov test, *D* = 0.221, *p* < 0.001). **G.** Left Panel: Histogram of RI of GLM-defined nondirectional delay-phase selective neurons in correct (orange), error (red open), and shuffled (gray) trials. Right Panel: CDF of the left histogram. Among nondirectional, delay phase-selective neurons, the distributions of RI values for correct and error trials were not significantly different (two-sample Kolmogorov–Smirnov test, *D* = 0.086, *p* = 0.562), suggesting minimal separation between the two conditions. **H.** Scatter plot comparing the RI (Correct) with the difference between RI (Incorrect) and RI (Correct) in nondirectional single-phase selective neurons within PPC. ΔRI values closer to −1 indicate that the response during incorrect trials was closer to the opposite direction compared to correct trials. **I.** Scatter plot comparing the RI (Correct) with the difference between RI (Incorrect) and RI (Correct) in nondirectional sample-phase selective neurons within PPC. **J.** Scatter plot comparing the RI (Correct) with the difference between RI (Incorrect) and RI (Correct) in nondirectional delay-phase selective neurons within PPC. Underlying data and analysis code for this figure are available at DOI: https://doi.org/10.5281/zenodo.15872574.
(TIFF)

S8 Fig The fraction of variance explained by principal components. **A.** The fraction of variance explained by principal components of right directional phase-specific neurons (bar) and the accumulated variance explained (line). **B.** Same as A, but for left-directional phase-specific neurons. **C.** Same analysis as in A and B, performed on the combined population of right- and left-directional phase-specific neurons. Underlying data and analysis code for this figure are available at DOI: https://doi.org/10.5281/zenodo.15872574.
(TIFF)

S9 Fig Time dependent ROC analysis of the trajectory and schematic of the trajectory similarity index (TSI). **A.** Example schematic for a rightward‐preferring neuron, comparing the trajectories of correct preferred–preferred (PP) versus correct opposite–opposite (OO) responses and illustrating the corresponding trajectories on correct and error trials. **B.** 50% of the correct trials were used to create a vector, and the remaining 50% were projected onto it, repeated over 100 random trials. ROC analysis was conducted on each distance value over time, with the *auROC* values indicating the following: values close to 0 suggest smaller differences in the compared distances, while values approaching −1 or 1 indicate larger differences. The blue line compares D0 and D3, while the red line compares D0 and D4. Values closer to −1 indicate a lower probability that D0 has a smaller value compared to D3 or D4. **C.** The blue line compares d1 and d2, while the red line compares d1 and d5. Values closer to 1 indicate a higher probability that d1 has a larger value compared to d2 or d5**. D.** At each time point, the trajectory distance from the trial-averaged values to the mean PP and the distance to the OO were measured to calculate the trajectory selectivity index, as shown in the formula. If the measured population activity at a given time is similar to the preferred correct response, the TSI will be positive. Conversely, activities similar to the nonpreferred correct response will result in a negative TSI value. Underlying data and analysis code for this figure are available at DOI: https://doi.org/10.5281/zenodo.15872574.
(TIFF)

S10 Fig Error-trial activation of opposite-direction neurons, contrasting with the preferred-direction neurons shown in Fig 5. A. Schematic of the population analysis approach used to quantify trajectory distances. Unlike Fig 5, which analyzed stimulus-preferred neurons (e.g., right-preferring neurons during right-stimulus trials), here we focus on neurons that prefer the opposite direction—specifically, right-preferring neurons during left-stimulus trials—which are expected to remain largely inactive when the stimulus is nonpreferred. **B.** Schematic illustrating trial structure for right-preferring neurons in left-stimulus trials: In OO trials (correct), the leftward stimulus does not match these neurons’ tuning, and thus they remain silent—establishing the expected response baseline. In OP trials (error), the stimulus remains leftward, but the animal responds rightward. Here, the right-preferring neurons become inappropriately active, signaling a drift toward the incorrect stimulus representation. PP trials (correct rightward stimulus and right-preferring neurons) serve as a reference for the canonical preferred-direction activity. **C.** Euclidean distance between population activity trajectories in OO versus OP (same-stimulation error). Despite identical sensory inputs, the divergence in activity during the delay period indicates aberrant activation of nonpreferred neurons during errors. **D.** Euclidean distance between PP and OP (same-response error). Underlying data and analysis code for this figure are available at DOI: https://doi.org/10.5281/zenodo.15872574.
(TIFF)

S11 Fig LDA‐based SNR time courses for same‐stimulation and same‐response errors across delay durations. Sessions were split by delay length (1.5 s vs. 2 s) and analyzed as in Fig 6B. In each session, a linear discriminant analysis classifier was trained on correct trials within a 50-frame sliding window (1-frame step) and then used to project incorrect trials, yielding a signal-to-noise ratio (SNR) for each window; these window-based SNR values were then averaged across sessions. **A.** SNR profile for same‐stimulation error trials, in which the stimulus on error trials matched that of correct trials. **B.** SNR profile for same‐response error trials, in which the licking direction on error trials matched that of correct trials. Underlying data and analysis code for this figure are available at DOI: https://doi.org/10.5281/zenodo.15872574.
(TIFF)

S12 Fig Trial-by-trial relationship between coding-direction distance and relative reaction time. **A.** Example session illustrating the analysis pipeline. The coding direction (CD) was defined using correct trials only: a linear decoder was trained on 50% of the trials (randomly selected) and applied to the remaining 50%. Population activity was aligned to motor onset, and the CD_delay vector was computed from the delay epoch (1.5 or 2 s). For each trial, its projection onto this CD vector was averaged over the 1-second window preceding motor onset, and its distance to the decision boundary—defined as the midpoint between mean CD projections of RR and LL trials—was calculated. An example trajectory of CD projection is highlighted in orange. Reaction time was measured as the interval from lick port movement onset to lick detection. While movement onset was precisely recorded, the duration of the servo motor movement could vary and was not reliably quantified. To account for this variability, we computed relative reaction time as each trial’s deviation from the session’s median RT. **B.** Scatter plot of pooled data across all sessions, showing trial-by-trial CD distance and relative reaction time. Pearson’s correlation analysis revealed no significant relationship (*r* = –0.031, *p* = 0.530), suggesting that variability in motor timing is not systematically related to delay-period coding strength. Underlying data and analysis code for this figure are available at DOI: https://doi.org/10.5281/zenodo.15872574.
(TIFF)

S13 Fig Effect of behavioral performance on population activity correlations during correct and error trials. **A.** Pearson’s correlation of population activity vectors within correct trials across time bins (each bin = 25 frames; bin: 21–45, 46–70, 71–95, 96–120, 121–145, and 146–170). For each session, activity vectors (neuron × time) from 50% of correct trials were randomly selected and correlated with the remaining 50%. Sessions were divided into two groups: high-performance (blue; correct rate >70%; *n* = 4 sessions) and low-performance (gray; correct rate ≤70%; *n* = 7 sessions). Correlation values were significantly higher in high-performance sessions at bins 2–3 and 5–6 (nonparametric unpaired *t* test, *p* = [0.0727, 0.0424, 0.0424, 0.1091, 0.0242, 0.0121]). **B.** Same as (A), but comparing correct trials and same-stimulation error trials. High-performance sessions (blue) exhibited a larger decline in correlation during the delay phase, suggesting that population-level representations diverge more strongly from correct-trial activity in better-trained sessions—potentially reflecting more sharply defined or more actively degraded sensory representations during error trials. No significant between-group difference was observed across bins (*p* = [0.1091, 0.1091, 0.3152, 0.6485, 0.9273, 0.7879]). A significant negative trend across time was detected only in the high-performance group (Spearman’s *ρ* = –1, *p* = 0.0028), but not in the low-performance group (*ρ* = –0.3143, *p* = 0.5639). Underlying data and analysis code for this figure are available at DOI: https://doi.org/10.5281/zenodo.15872574.
(TIFF)

S14 Fig Higher decoding accuracy during the delay phase in high-performance sessions. **A.** Prediction accuracy during the delay phase (frames 121–170) for each session, color-coded by behavioral performance. An SVM classifier was trained to distinguish correct-right versus correct-left trials using an 8-frame sliding window (step size: 1 frame). For each session, 70% of trials were randomly selected for training and 30% for testing, repeated 10 times to compute mean decoding accuracy. Sessions with higher behavioral performance (warmer colors) exhibited consistently greater decoding accuracy throughout the delay period. **B.** Mean decoding accuracy across the delay phase, plotted by session and grouped by performance level (good: correct rate >70%; low: < 70%). High-performance sessions showed significantly higher decoding accuracy than low-performance sessions (nonparametric unpaired *t* tes*t*, *p* = 0.006). Underlying data and analysis code for this figure are available at DOI: https://doi.org/10.5281/zenodo.15872574.
(TIFF)

S15 Fig Framework of the proposed method for automatic event-related ROI detection. **A.** The proposed method detects an event-related neuron pixelwise. **B.** Pixelwise error optimization with amplitude of calcium signal *β*. **C.** The 1-sampled *t* test is applied to the *β* distribution of each pixel. **D.** Selected ROI pixels are clustered automatically.
(TIFF)

## Abbreviations

CD: coding direction
CDF: cumulative distribution function
GLM: generalized linear model
LDA: linear discriminant analysis
PCA: principal component analysis
PI: preference index
PP: preferred–preferred
PPC: posterior parietal cortex
PSI: phase selective index
RI: reliability index
ROC: receiver operating characteristic
ROI: region of interest
RT: reaction time
SNR: signal-to-noise ratio
STM: short-term memory
SVM: support vector machine
TSI: trajectory similarity index
