# Supplementary figures and images for: Short-term memory errors are strongly associated with a drift in neural activity in the posterior parietal cortex
Source: PLoS Biol. 2025 Sep 3;23(9):e3003359. doi: 10.1371/journal.pbio.3003359 (PMC12435700; doi:10.1371/journal.pbio.3003359)

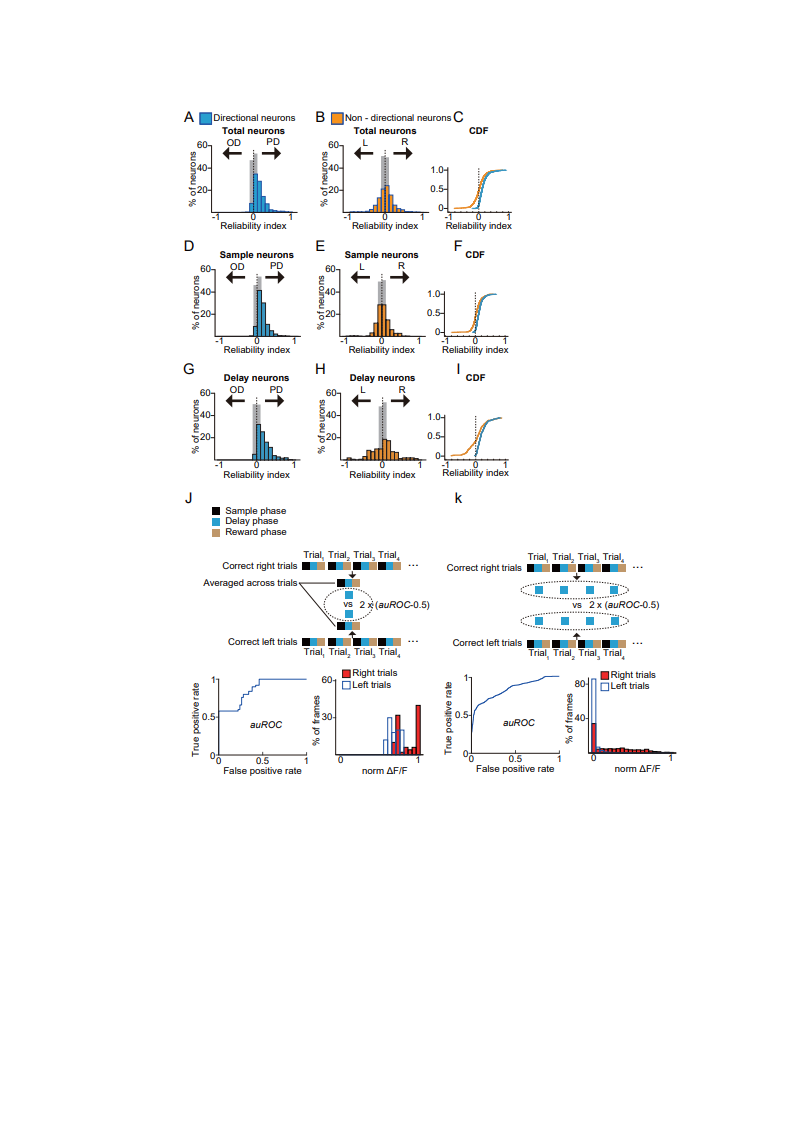

Supplement: S4 Fig — A. Histogram of reliability indices (RI) for GLM-defined all directional single-phase specific neurons in correct trials (light blue), and shuffled trials (gray). B. Histogram of RI for GLM-defined all nondirectional single-phase specific neurons in correct trials (orange), and shuffled trials (gray). C. Cumulative distribution function (CDF) of variable for directional single-phase specific neurons (light blue line) and nondirectional single-phase specific neurons (orange line). The distributions of RI values for directional and nondirectional neurons were significantly different (two-sample Kolmogorov–Smirnov test, D = 0.379, p < 0.001). D. Histogram of RI for GLM-defined directional sample-phase specific neurons in correct trials (light blue), and shuffled trials (gray). E. Histogram of RI for GLM-defined nondirectional sample-phase specific neurons in correct trials (orange), and shuffled trials (gray). F. CDF of variable for directional sample-phase specific neurons (light blue line) and nondirectional sample-phase specific neurons (orange line). Among sample phase-selective neurons, the distributions of RI values for directional and nondirectional neurons were significantly different (two-sample Kolmogorov–Smirnov test, D = 0.369, p < 0.001). G. Histogram of RI for GLM-defined directional delay-phase specific neurons in correct trials (light blue), and shuffled trials (gray). H. Histogram of RI for GLM-defined nondirectional delay-phase specific neurons in correct trials (orange), and shuffled trials (gray). I. CDF of variable for directional delay-phase specific neurons (light blue line) and nondirectional delay -phase specific neurons (orange line). Among delay phase-selective neurons, the distributions of RI values for directional and nondirectional neurons were significantly different (two-sample Kolmogorov–Smirnov test, D = 0.378, p < 0.001). J. Schematic illustration of PI calculation. Trial-aligned calcium activity from correct rightward and leftward tr [file pbio.3003359.s004.tiff]

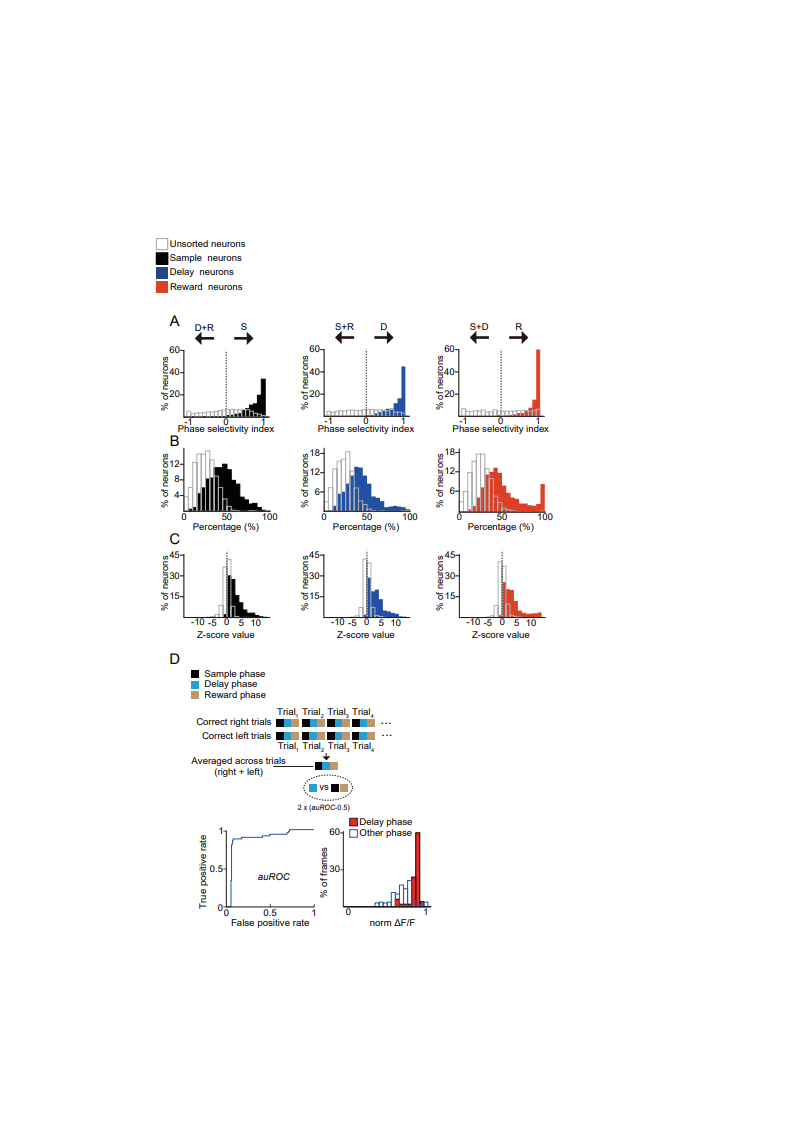

Supplement: S5 Fig — A. Left Panel: histogram of phase selectivity indices (PSI) for GLM-defined sample-phase specific neurons in correct trials (light blue), and unsorted neurons in correct trials (gray). A more positive phase selectivity index indicates a more specific response to the corresponding phase. Middle Panel: histogram of PSI for GLM-defined delay-phase specific neurons in correct trials (light blue), and unsorted neurons in correct trials (gray). Right Panel: histogram of PSI for GLM-defined reward-phase specific neurons in correct trials (light blue), and unsorted neurons in correct trials (gray). B. Permutation-based validation of epoch-specific neuron sorting. To assess the accuracy of our classification, we computed each neuron’s mean activity on every trial during the Sample, Delay, and Reward epochs, then generated null Gaussian distributions for each epoch by randomly shuffling trial labels 1,000 times. For each epoch, we calculated the percentage of trials in which the neuron’s observed mean activity exceeded the one-tailed p < 0.05 threshold of the corresponding null distribution—an x-axis value of 100% therefore indicates that the neuron showed statistically significant, epoch-specific activity on every trial. C. Using the same permutation procedure as in Fig 5B, null Gaussian distributions were generated for each epoch. For each neuron, we then calculated its single mean activity across all trials in the epoch of interest and expressed its position within the corresponding null distribution as a Z-score. Higher Z-score values therefore, indicate stronger epoch-specific modulation of the neuron’s activity. D. Schematic illustration of Phase Selectivity Index (PSI) calculation for a task-phase–selective neuron. Neuronal activity from correct rightward and leftward trials is pooled and aligned to each task phase: sample (black), delay (blue), and reward (tan). To quantify phase selectivity, normalized ΔF/F signals during the phase of interest (e.g., the delay phase [file pbio.3003359.s005.tiff]

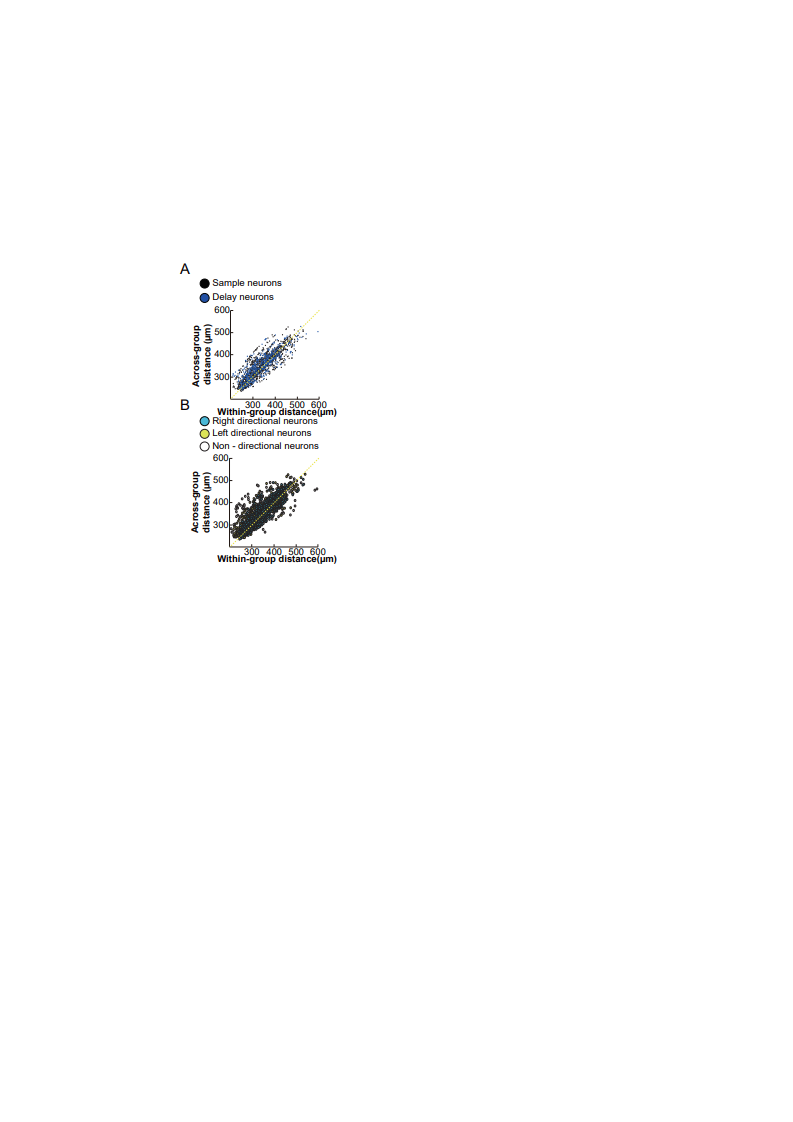

Supplement: S6 Fig — A. The relationship between within-group and across-group distances for phase-selective neurons. B. The within-group and across-group distances for directional and non-directional neurons. Underlying data and analysis code for this figure are available at DOI: https://doi.org/10.5281/zenodo.15872574. (TIFF) [file pbio.3003359.s006.tiff]

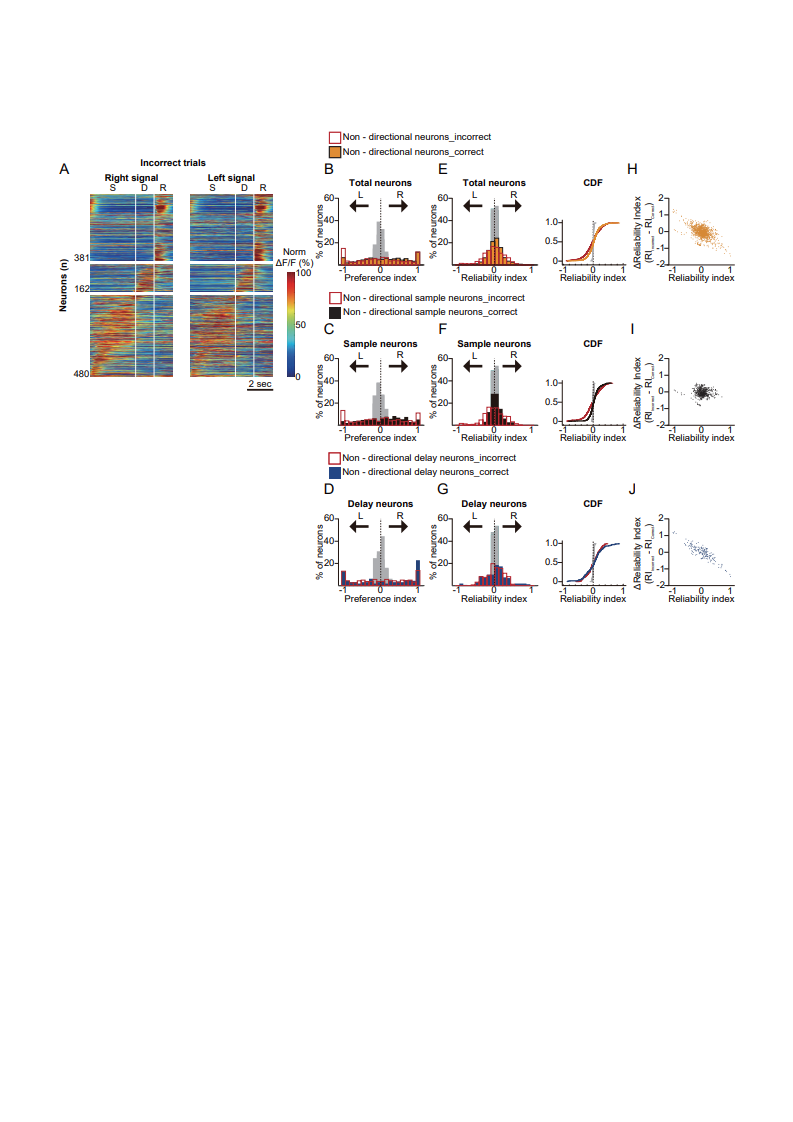

Supplement: S7 Fig — A. Color-coded trial average ΔF/F of nondirectional single-phase selective neurons in error trials. ΔF/F was normalized to the maximum ΔF/F in correct trials and aligned based on the time-to-peak of each neuron. B. Histogram of PI of GLM-defined nondirectional single-phase selective neurons in correct (orange), error (red open), and shuffled (gray) trials. C. Histogram of PI of GLM-defined nondirectional sample-phase selective neurons in correct (black), error (red open), and shuffled (gray) trials. D. Histogram of PI of GLM-defined nondirectional delay-phase selective neurons in correct (blue), error (red open), and shuffled (gray) trials. E. Left Panel: Histogram of RI of GLM-defined nondirectional single-phase selective neurons in correct (orange), error (red open), and shuffled (gray) trials. Right Panel: CDF of the left histogram. Among nondirectional neurons, the distributions of RI values for correct and error trials were significantly different (two-sample Kolmogorov–Smirnov test, D = 0.132, p < 0.001), albeit with a smaller effect size compared to directional neurons. F. Left Panel: Histogram of RI of GLM-defined nondirectional sample-phase selective neurons in correct (orange), error (red open), and shuffled (gray) trials. Right Panel: CDF of the left histogram. Among nondirectional, sample phase-selective neurons, the distributions of RI values for correct and error trials were significantly different (two-sample Kolmogorov–Smirnov test, D = 0.221, p < 0.001). G. Left Panel: Histogram of RI of GLM-defined nondirectional delay-phase selective neurons in correct (orange), error (red open), and shuffled (gray) trials. Right Panel: CDF of the left histogram. Among nondirectional, delay phase-selective neurons, the distributions of RI values for correct and error trials were not significantly different (two-sample Kolmogorov–Smirnov test, D = 0.086, p = 0.562), suggesting minimal separation between the two conditions. H. Scatter plot comparing the RI (Correct) [file pbio.3003359.s007.tiff]

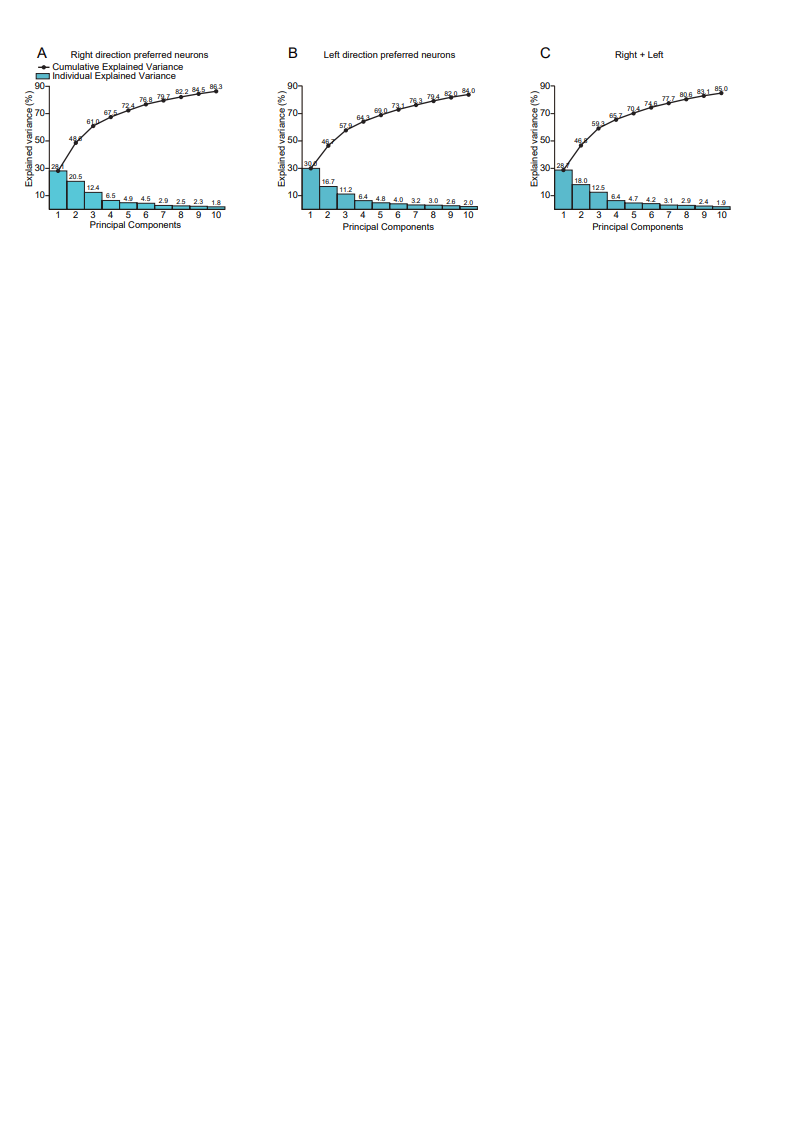

Supplement: S8 Fig — A. The fraction of variance explained by principal components of right directional phase-specific neurons (bar) and the accumulated variance explained (line). B. Same as A, but for left-directional phase-specific neurons. C. Same analysis as in A and B, performed on the combined population of right- and left-directional phase-specific neurons. Underlying data and analysis code for this figure are available at DOI: https://doi.org/10.5281/zenodo.15872574. (TIFF) [file pbio.3003359.s008.tiff]

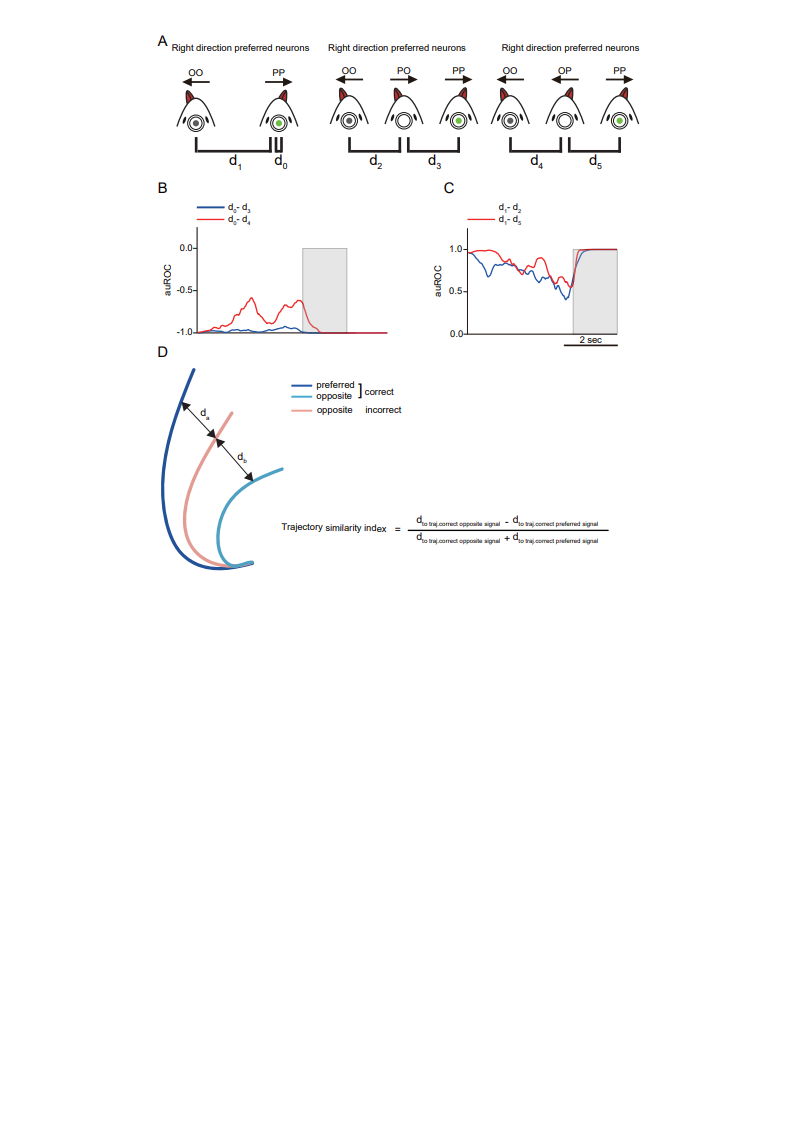

Supplement: S9 Fig — A. Example schematic for a rightward‐preferring neuron, comparing the trajectories of correct preferred–preferred (PP) versus correct opposite–opposite (OO) responses and illustrating the corresponding trajectories on correct and error trials. B. 50% of the correct trials were used to create a vector, and the remaining 50% were projected onto it, repeated over 100 random trials. ROC analysis was conducted on each distance value over time, with the auROC values indicating the following: values close to 0 suggest smaller differences in the compared distances, while values approaching −1 or 1 indicate larger differences. The blue line compares D0 and D3, while the red line compares D0 and D4. Values closer to −1 indicate a lower probability that D0 has a smaller value compared to D3 or D4. C. The blue line compares d1 and d2, while the red line compares d1 and d5. Values closer to 1 indicate a higher probability that d1 has a larger value compared to d2 or d5. D. At each time point, the trajectory distance from the trial-averaged values to the mean PP and the distance to the OO were measured to calculate the trajectory selectivity index, as shown in the formula. If the measured population activity at a given time is similar to the preferred correct response, the TSI will be positive. Conversely, activities similar to the nonpreferred correct response will result in a negative TSI value. Underlying data and analysis code for this figure are available at DOI: https://doi.org/10.5281/zenodo.15872574. (TIFF) [file pbio.3003359.s009.tiff]

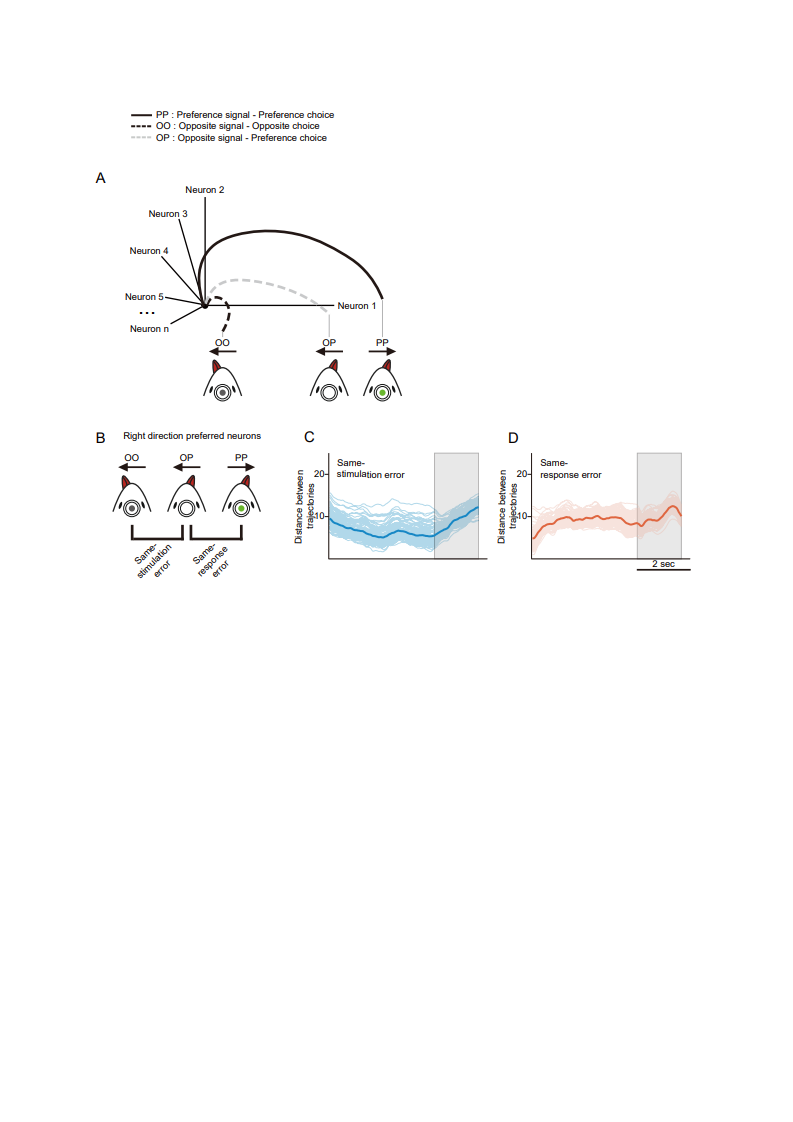

Supplement: S10 Fig — A. Schematic of the population analysis approach used to quantify trajectory distances. Unlike Fig 5, which analyzed stimulus-preferred neurons (e.g., right-preferring neurons during right-stimulus trials), here we focus on neurons that prefer the opposite direction—specifically, right-preferring neurons during left-stimulus trials—which are expected to remain largely inactive when the stimulus is nonpreferred. B. Schematic illustrating trial structure for right-preferring neurons in left-stimulus trials: In OO trials (correct), the leftward stimulus does not match these neurons’ tuning, and thus they remain silent—establishing the expected response baseline. In OP trials (error), the stimulus remains leftward, but the animal responds rightward. Here, the right-preferring neurons become inappropriately active, signaling a drift toward the incorrect stimulus representation. PP trials (correct rightward stimulus and right-preferring neurons) serve as a reference for the canonical preferred-direction activity. C. Euclidean distance between population activity trajectories in OO versus OP (same-stimulation error). Despite identical sensory inputs, the divergence in activity during the delay period indicates aberrant activation of nonpreferred neurons during errors. D. Euclidean distance between PP and OP (same-response error). Underlying data and analysis code for this figure are available at DOI: https://doi.org/10.5281/zenodo.15872574. (TIFF) [file pbio.3003359.s010.tiff]

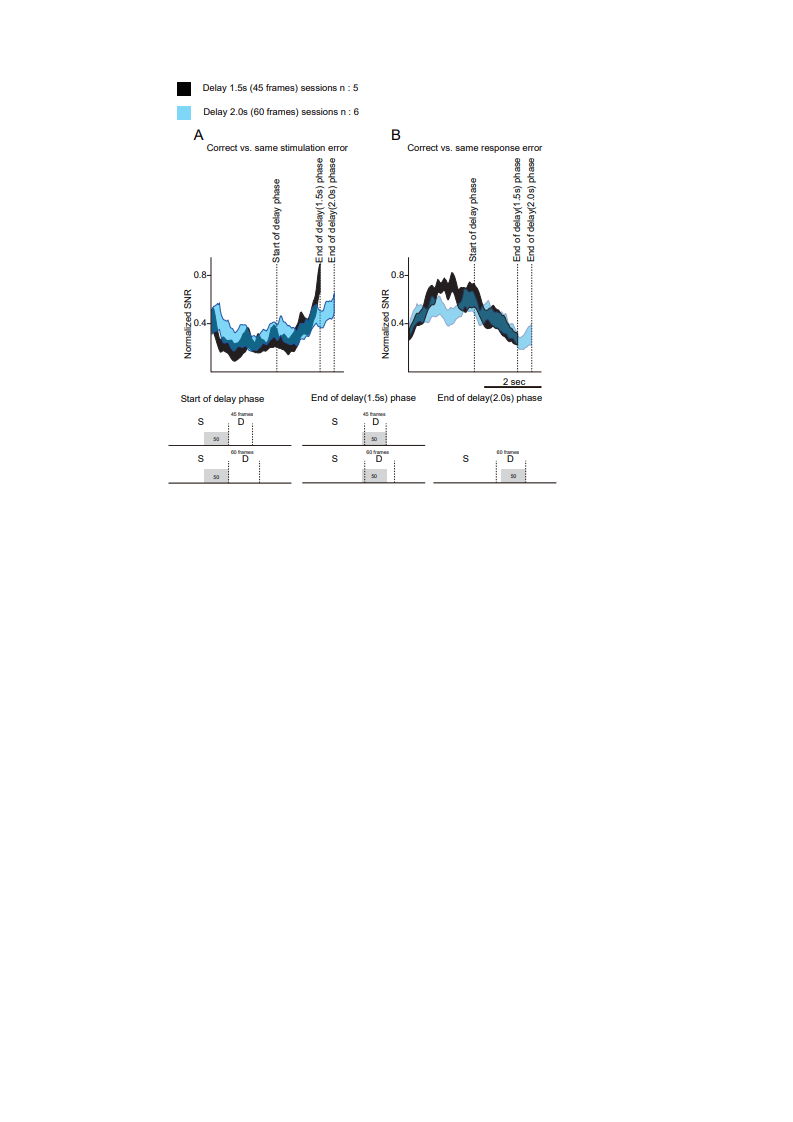

Supplement: S11 Fig — Sessions were split by delay length (1.5 s vs. 2 s) and analyzed as in Fig 6B. In each session, a linear discriminant analysis classifier was trained on correct trials within a 50-frame sliding window (1-frame step) and then used to project incorrect trials, yielding a signal-to-noise ratio (SNR) for each window; these window-based SNR values were then averaged across sessions. A. SNR profile for same‐stimulation error trials, in which the stimulus on error trials matched that of correct trials. B. SNR profile for same‐response error trials, in which the licking direction on error trials matched that of correct trials. Underlying data and analysis code for this figure are available at DOI: https://doi.org/10.5281/zenodo.15872574. (TIFF) [file pbio.3003359.s011.tiff]

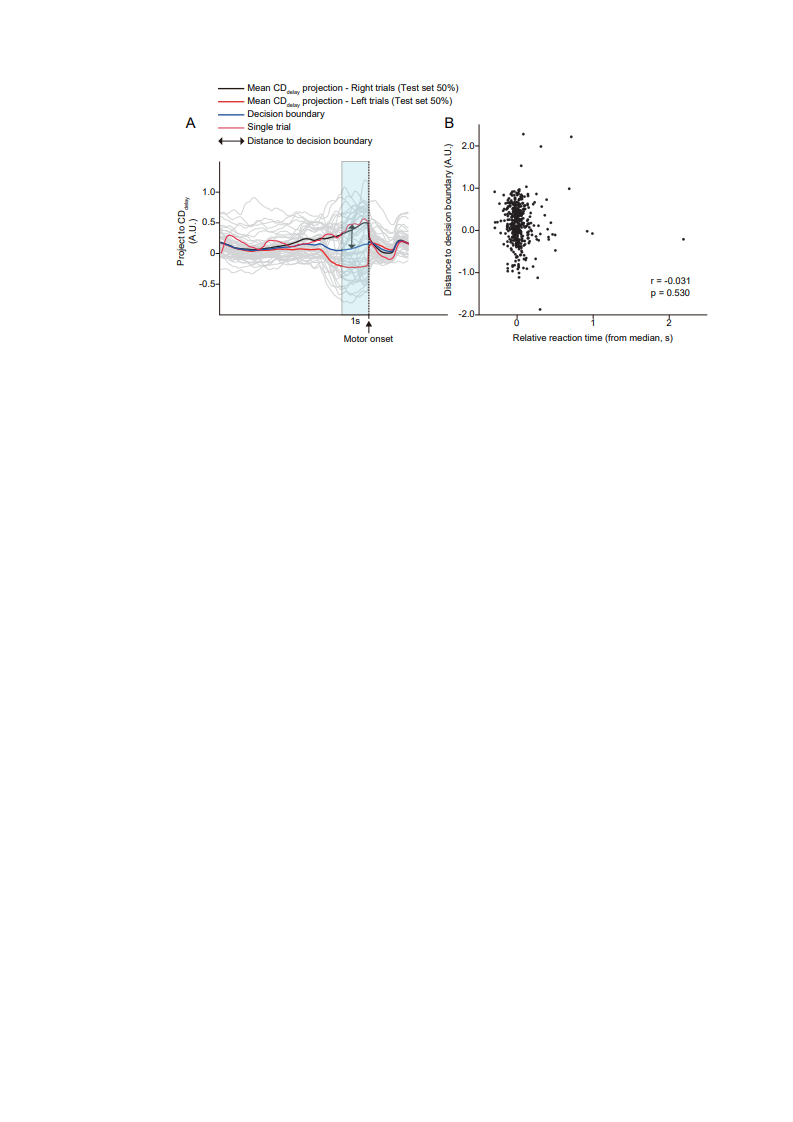

Supplement: S12 Fig — A. Example session illustrating the analysis pipeline. The coding direction (CD) was defined using correct trials only: a linear decoder was trained on 50% of the trials (randomly selected) and applied to the remaining 50%. Population activity was aligned to motor onset, and the CD_delay vector was computed from the delay epoch (1.5 or 2 s). For each trial, its projection onto this CD vector was averaged over the 1-second window preceding motor onset, and its distance to the decision boundary—defined as the midpoint between mean CD projections of RR and LL trials—was calculated. An example trajectory of CD projection is highlighted in orange. Reaction time was measured as the interval from lick port movement onset to lick detection. While movement onset was precisely recorded, the duration of the servo motor movement could vary and was not reliably quantified. To account for this variability, we computed relative reaction time as each trial’s deviation from the session’s median RT. B. Scatter plot of pooled data across all sessions, showing trial-by-trial CD distance and relative reaction time. Pearson’s correlation analysis revealed no significant relationship (r = –0.031, p = 0.530), suggesting that variability in motor timing is not systematically related to delay-period coding strength. Underlying data and analysis code for this figure are available at DOI: https://doi.org/10.5281/zenodo.15872574. (TIFF) [file pbio.3003359.s012.tiff]

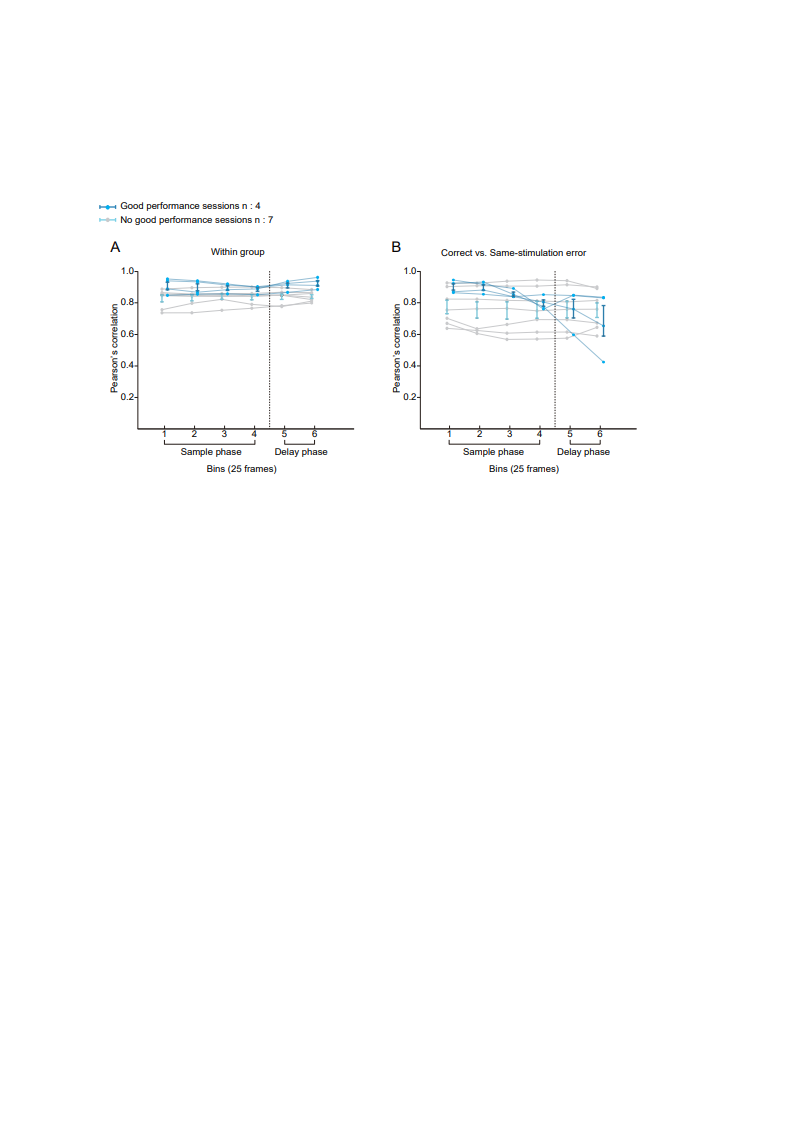

Supplement: S13 Fig — A. Pearson’s correlation of population activity vectors within correct trials across time bins (each bin = 25 frames; bin: 21–45, 46–70, 71–95, 96–120, 121–145, and 146–170). For each session, activity vectors (neuron × time) from 50% of correct trials were randomly selected and correlated with the remaining 50%. Sessions were divided into two groups: high-performance (blue; correct rate >70%; n = 4 sessions) and low-performance (gray; correct rate ≤70%; n = 7 sessions). Correlation values were significantly higher in high-performance sessions at bins 2–3 and 5–6 (nonparametric unpaired t test, p = [0.0727, 0.0424, 0.0424, 0.1091, 0.0242, 0.0121]). B. Same as (A), but comparing correct trials and same-stimulation error trials. High-performance sessions (blue) exhibited a larger decline in correlation during the delay phase, suggesting that population-level representations diverge more strongly from correct-trial activity in better-trained sessions—potentially reflecting more sharply defined or more actively degraded sensory representations during error trials. No significant between-group difference was observed across bins (p = [0.1091, 0.1091, 0.3152, 0.6485, 0.9273, 0.7879]). A significant negative trend across time was detected only in the high-performance group (Spearman’s ρ = –1, p = 0.0028), but not in the low-performance group (ρ = –0.3143, p = 0.5639). Underlying data and analysis code for this figure are available at DOI: https://doi.org/10.5281/zenodo.15872574. (TIFF) [file pbio.3003359.s013.tiff]

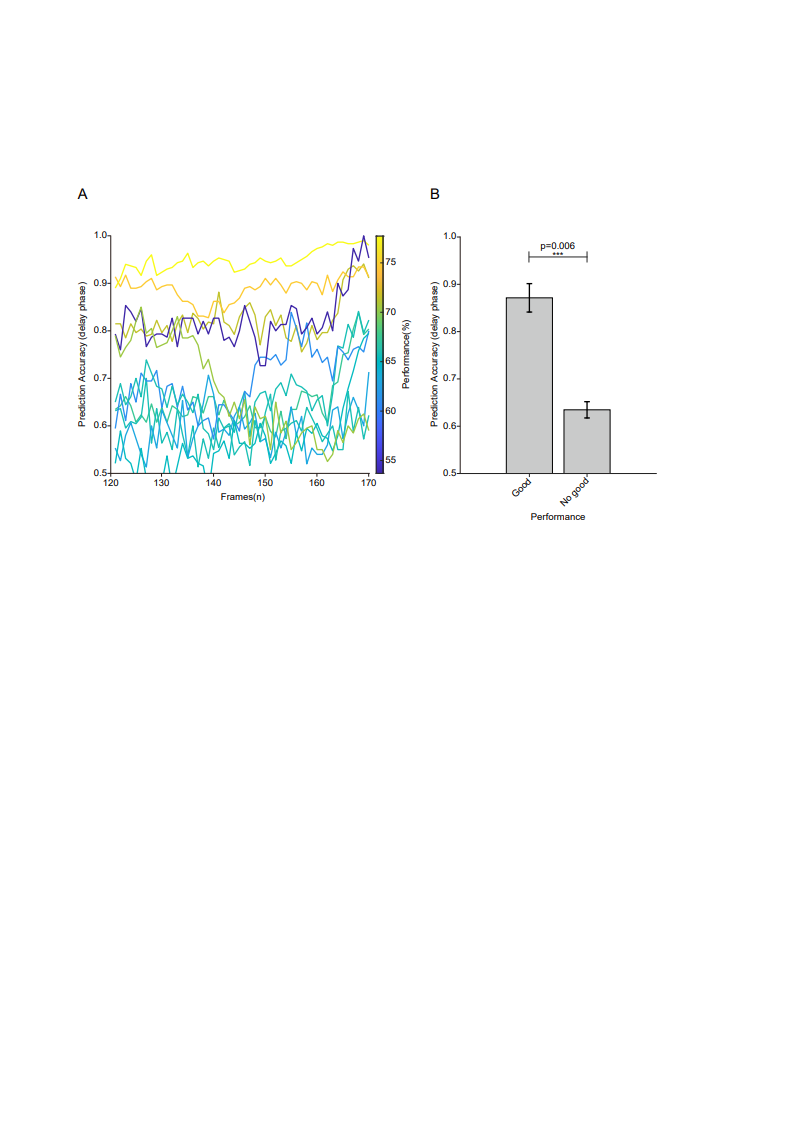

Supplement: S14 Fig — A. Prediction accuracy during the delay phase (frames 121–170) for each session, color-coded by behavioral performance. An SVM classifier was trained to distinguish correct-right versus correct-left trials using an 8-frame sliding window (step size: 1 frame). For each session, 70% of trials were randomly selected for training and 30% for testing, repeated 10 times to compute mean decoding accuracy. Sessions with higher behavioral performance (warmer colors) exhibited consistently greater decoding accuracy throughout the delay period. B. Mean decoding accuracy across the delay phase, plotted by session and grouped by performance level (good: correct rate >70%; low: < 70%). High-performance sessions showed significantly higher decoding accuracy than low-performance sessions (nonparametric unpaired t test, p = 0.006). Underlying data and analysis code for this figure are available at DOI: https://doi.org/10.5281/zenodo.15872574. (TIFF) [file pbio.3003359.s014.tiff]

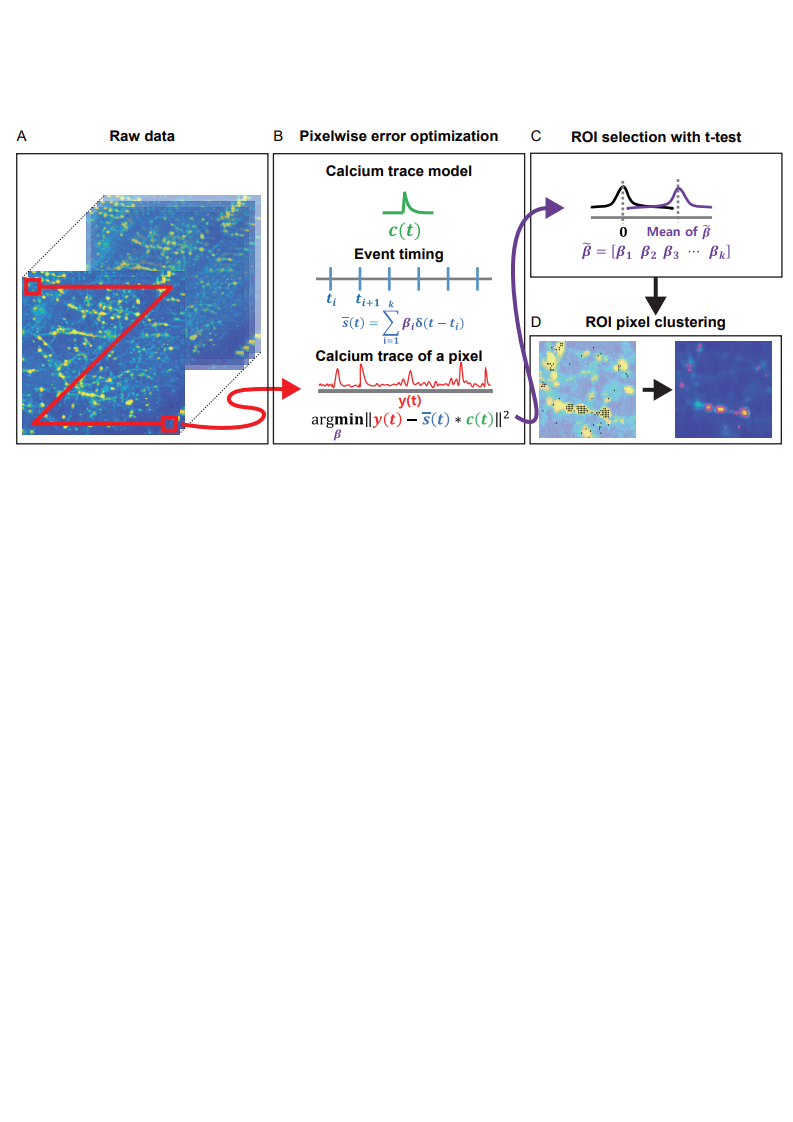

Supplement: S15 Fig — A. The proposed method detects an event-related neuron pixelwise. B. Pixelwise error optimization with amplitude of calcium signal β. C. The 1-sampled t test is applied to the β distribution of each pixel. D. Selected ROI pixels are clustered automatically. (TIFF) [file pbio.3003359.s015.tiff]
